# Geometric Constraints on Human Speech Sound Inventories

**DOI:** 10.3389/fpsyg.2016.01061

**Published:** 2016-07-12

**Authors:** Ewan Dunbar, Emmanuel Dupoux

**Affiliations:** Laboratoire de Sciences Cognitives et Psycholinguistique (ENS–EHESS–Centre National de la Recherche Scientifique), Département des Études Cognitives, École Normale Supérieure–PSL Research UniversityParis, France

**Keywords:** phonology, phonetics, language typology, sound inventories, feature economy

## Abstract

We investigate the idea that the languages of the world have developed coherent sound systems in which having one sound increases or decreases the chances of having certain other sounds, depending on shared properties of those sounds. We investigate the geometries of sound systems that are defined by the inherent properties of sounds. We document three typological tendencies in sound system geometries: economy, a tendency for the differences between sounds in a system to be definable on a relatively small number of independent dimensions; local symmetry, a tendency for sound systems to have relatively large numbers of pairs of sounds that differ only on one dimension; and global symmetry, a tendency for sound systems to be relatively balanced. The finding of economy corroborates previous results; the two symmetry properties have not been previously documented. We also investigate the relation between the typology of inventory geometries and the typology of individual sounds, showing that the frequency distribution with which individual sounds occur across languages works in favor of both local and global symmetry.

## 1. Introduction

Typological studies of human languages have helped give insight into language cognition. For example, the fact that certain sequences of sounds are more common in the world's languages than others (sequences like *pliff* are more common than sequences like *lpiff*) suggests that these sequences may put less inherent load on the speech perception and/or speech production systems, a hypothesis that has been corroborated using other sources of evidence (Ohala, 1999; Berent et al., 2007). Other cognitive restrictions supported by language typology are computational limitations on how the syntactic and semantic systems work to combine meaningful units (Stabler, 2013), and on how the phonetic and phonological systems are used by listeners to regulate what patterns of sounds are perceived as natural in a language (Heinz, 2016).

Here, we investigate the intuition that languages do not make up words and sentences by haphazardly selecting from a set of possible speech sounds (segments, like the [p] sound in English *spit*, the [i] sound in *fee*, and so on). Rather, languages are made up of coherent *sound systems* in which the presence of one sound takes into account the existence of the other sounds. Two major ideas have been formulated in the literature regarding constraints on natural sound systems. The first one, dispersion theory, suggests a causative role for efficient communication. It proposes that sound systems attempt to simultaneously minimize articulatory effort, while maximizing the perceptual distinctiveness of contrasts and the rate of transmission of information (Liljencrants and Lindblom, 1972; Flemming, 2001; Vaux and Samuels, 2015). The other idea is that sound systems tend to make efficient, or economical, use of the dimensions that define sounds in the sounds in the human speech perception systems, speech motor systems, and/or lexical storage (word memory) systems (Martinet, 1939; Clements, 2003). We expand on the second line of research, casting it in terms of *geometric properties* of sound systems, introducing two new properties besides the original notion of economy and measuring them on a large collection of languages.

Linguists describe sounds in terms of discrete dimensions, or features, which enable them to define sound classes (like vowels, consonants, nasals, stops, and so on), as well as to define relations between sounds (for instance, [p] and [b] differ in the feature *voicing*; so do [t] and [d], [k] and [ɡ], and so on). These features enable us to characterize segment inventories in geometric terms, according to their position in a hypercube.

We document three typological tendencies in the geometries of sound inventories across the world's languages: *economy, local symmetry*, and *global symmetry* (**Study 1**). The finding of economy corroborates previous results. The two symmetry properties have not been previously documented: the number of pairs like [p]–[b], which differ *only* in one feature, and the level of balance overall in the inventory between, for example, voiced and voiceless sounds (and so on for other features), are both much higher than we should expect by chance. **Study 2** investigates the relation between the typology of inventory geometries and the typology of individual sounds. It shows that the frequency distribution of individual sounds across the world's languages works in favor of local symmetry and global symmetry.

## 2. Characterizing inventory geometries

**Figure 1:**
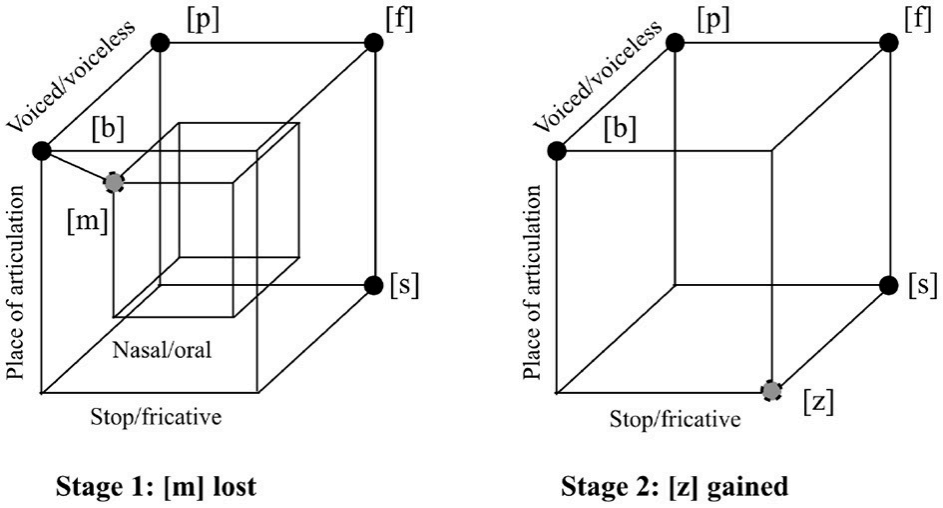
**Two stages in the history of a hypothetical language, illustrating the notion of economy as a geometric property of sound systems**. Four idealized articulatory parameters are shown: place of articulation ([b], [p], [f], and [m] are made with constriction at the lips, while [s] and [z] are made by placing the tongue just above and behind the upper teeth); voicing ([b], [m], and [z] are made with vibration of the vocal cords, while [f] and [s] are without); nasal vs. oral airflow ([m] is made with the velum lowered so that air passes through the nose, while the other sounds are made with the velum raised, allowing airflow only in the mouth); and stop vs. fricative constriction ([b], [p], and [m] are made by totally blocking the flow of air through the mouth, while [f], [s], and [z] allow air to pass through a narrow opening and create noise). At Stage 1, the hypothetical language loses [m], increasing economy by eliminating one of the articulatory degrees of freedom of the inventory. At Stage 2, the language gains [z], increasing economy by making more use of the three remaining dimensions. The hypercube shown is a graph where the edges marked are between pairs of sounds of distance one in an idealized binary articulatory space. The remaining unsaturated edges of the interior and exterior cubes have also been added for clarity.

The geometric properties of an inventory are those properties that can be stated in terms of the relations between the sounds in the inventory, abstracting away from what the actual sounds are. Figure 1 illustrates the geometric notion of *economy*, proposed by Martinet (1939) as a property influencing how inventories change over time. When a language loses, gains, or changes the pronounciation of a sound, the result may make more efficient, or economical, use of the dimensions on which sounds vary in the inventory. In Figure 1, the hypothetical language loses [m], making all remaining consonants oral rather than nasal, removing all variability on the nasal/oral dimension. Although the number of sounds is smaller overall, it is now bigger relative to the number of distinct sounds that can be generated on these remaining three dimensions of variability (lips vs. tongue tip as place of articulation, voicing, stop vs. fricative: eight possible segments). The hypothetical inventory might then gain [z], which can be produced by varying only these three dimensions, making still better use of them.

Martinet's claim that economy characterizes the historical trajectory of language change predicts a typological tendency toward greater economy in inventories. A typological investigation by Clements (2003) corroborated this within small subsets of consonant inventories, and a more systematic study by Mackie and Mielke (2011) demonstrated greater than chance economy in a large number of languages, across the whole inventory (consonants and vowels together).

Economy is not the only geometric property of an inventory. Many geometric properties of sound systems were discussed by Trubetzkoy (1939), but the implicit claim that these were empirically demonstrable properties of natural sound inventories was never shown in any systematic typological survey. Some other typologically causal forces with implications for geometry were proposed, and some indirect predictions for inventory typology assessed, by Clements (2009).

We propose as exploratory measures two simple geometric properties of an inventory that can be directly measured: local and global symmetry. Local symmetry looks at a basic property of an inventory: the pairs of sounds that differ only in one dimension. These are what Trubetzkoy called “oppositions.” Trubetzkoy proposed many higher order properties of the oppositions in a system; local symmetry simply assesses whether the number of oppositions in a system, *N*_*mp*_, is relatively low or relatively high. For example, a vowel inventory in which sounds vary on four feature dimensions might have eight sounds (out of a possible 16). All such inventories would be equally economical, but, independent of their economy, they could have values of *N*_*mp*_ ranging between four and ten (it is geometrically impossible to arrange eight segments on four dimensions in such a way as to obtain more than ten oppositions, and if there were fewer than four, it would logically have to be the case that one of the feature dimensions was redundant: see Section 3.1 below).

**Figure 2:**
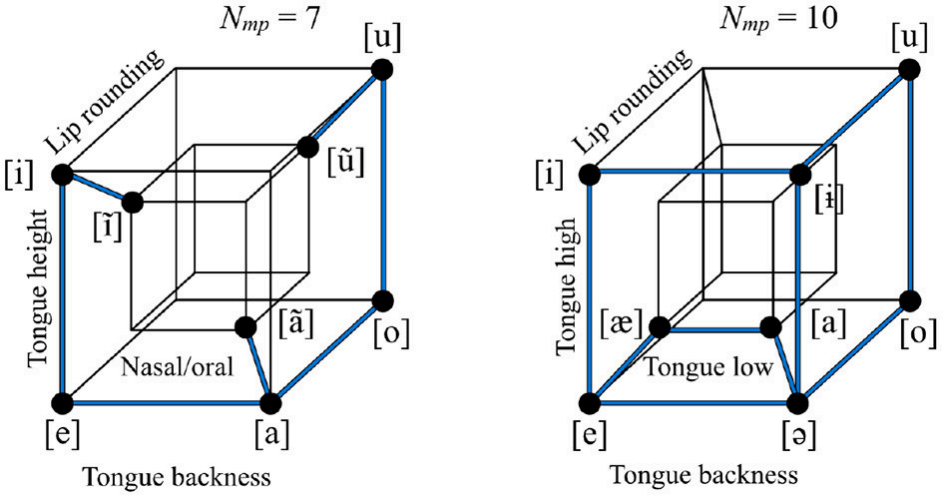
**The vowel inventories of Umbundu (left) vs. Bukiyip (right)**. The two inventories have the same size and number of non-redundant features (and therefore degree of economy), but differ in how many oppositions they have, that is, pairs of sounds that are distinguished only by one feature dimension, shown as dark blue lines (seven vs. ten). The vowel inventory of Bukiyip thus has higher local symmetry than that of Umbundu.

The vowel inventory of Umbundu (Niger-Congo, Angola) has lower local symmetry than the vowel inventory of Bukiyip (Torricelli, Papua New Guinea), as shown in Figure 2, where each opposition is shown as a dark blue edge on a hypercube. In an idealized space in which each dimension has only two possible values, the distance between two sounds can be defined as the number of dimensions on which the two sounds are different (the rectilinear or “city block” distance in that space). The local symmetry of an inventory is defined in terms of the total number of sound pairs with distance equal to 1, or, equivalently, the number of occupied edges in the hypercube.

**Figure 3:**
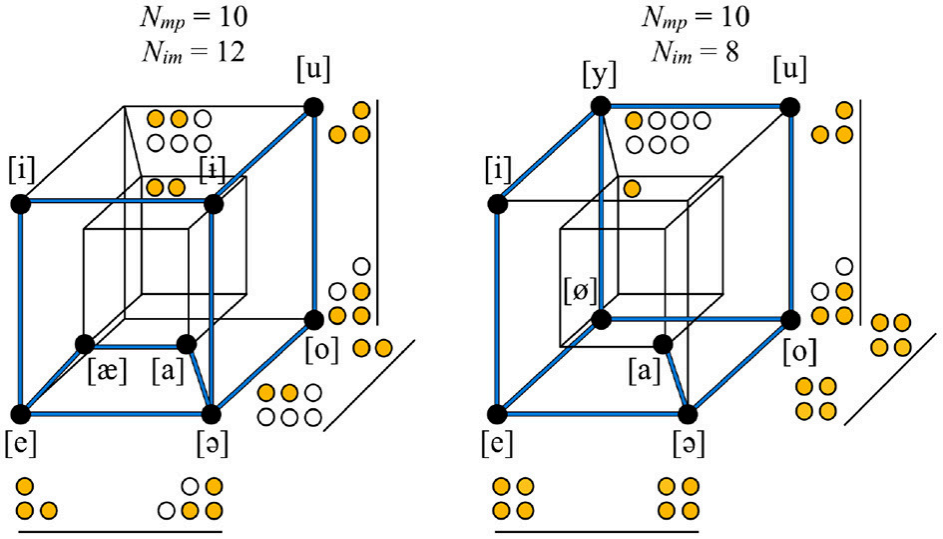
**The vowel inventories of Bukiyip (left) vs. Eastern Mari (right)**. Feature dimensions are the same as in the right panel of Figure 2. The two inventories have the same size, number of non-redundant features, economy, and number of oppositions, but the level of imbalance in their distribution throughout the space differs. For each dimension, the number of sounds with each of the two values is counted using filled or empty circles; the empty circles represent the sounds that tip the balance of the given dimension toward being less equally distributed between [+] and [−]. The total number of empty circles is less in Eastern Mari than in Bukiyip, and, thus Eastern Mari has greater global symmetry.

Global symmetry measures how well-balanced an inventory is on all of the feature dimensions on which it varies: whether all of the “mass” is distributed equally along all of the dimensions, or whether it is concentrated, like a loaded die, in a subpart of the hypercube. The level of imbalance can be measured by taking each non-redundant feature, finding the absolute difference in the number of sounds with the value [+] vs. [−], and then summing across all dimensions (*N*_*im*_). The value of *N*_*im*_ may be relatively low (well-balanced) or relatively high (imbalanced) for an inventory, given its other properties. For example, a vowel inventory with four non-redundant feature dimensions with eight sounds and ten oppositions could have its sounds distributed in various different ways throughout the space, yielding logically possible *N*_*im*_ values of twelve, ten, or eight, leading to progressively lower overall asymmetry, or, alternatively put, greater global symmetry. Figure 3 shows that Eastern Mari (Uralic, Russia) has greater global symmetry than Bukiyip.

Figure 2 shows inventories defined on two different sets of features (Umbundu is shown with a nasal/oral contrast where Bukiyip is shown with a low/non-low contrast). Inventories may differ in what features they vary on; for example, Bukiyip does not have a distinction between oral and nasal vowels. The notion of economy crucially relies on knowing how many features are “contrastive” for a given inventory. For the purposes of this study, we also define *N*_*mp*_ with respect to contrastive features, as well as *N*_*im*_. We discuss this further below in Section 3.1.

The property of having a large number of oppositions is not the same as having a high level of economy. However, the two are not independent. For example, any inventory with eight vowels, specifiable using three features (which would be fully economical) would necessarily have exactly *N*_*mp*_ equal to 12, with respect to those three features. There is, in fact, only one geometry over eight elements on three binary dimensions. That means that there is also only one possible value for *N*_*im*_ (namely, zero). It is also the case that *N*_*im*_ and *N*_*mp*_ are themselves not independent. We thus construct measures of them in which we decorrelate the three properties.

The quantitative measures we use in the present study crucially depend on a particular representation (feature system) for sounds (quite apart from the fact that, within that set of features, there may be various possibilities for which are contrastive). The measures we use are therefore not theory neutral; linguists and other speech scientists still debate the correct theory of cognitive representation for speech sounds, and it may differ depending on whether motor, perceptual, or lexical storage representations are relevant. The representation we used was one for which there existed a large annotated database of sound systems, consisting of 23 featural dimensions providing idealized articulatory descriptions for the inventories of the database of languages. It is a slight adaptation of one of the six feature systems used by Mielke et al. (2011) (in turn a small adaptation of the feature system of Halle and Clements, 1983), showing that the representation is good at describing sets of sounds that commonly occur in the sound patterns of the world's languages. It is very similar to, but not exactly the same as, the system (Chomsky and Halle, 1968) used by Mackie and Mielke (2011) to show robust effects of economy in natural inventories in the same database we use here (see below under **Study 1: Materials and Methods**; the full table for all the sounds appearing in the natural inventories in our sample is given in the Supplementary Materials).

Bearing these limitations in mind, our study does not aim at establishing absolute numbers for economy or symmetry, which would be not extremely telling, and might be very dependent on the representation used. Rather, our aim is to compare the geometrical properties of natural inventories with several types of random control inventories in such a way that the comparison will be resistant to notational or representational changes in the underlying feature system, since distortions to geometries introduced by our articulatory model will affect both natural and control inventories. Some geometric difference between natural and control inventories should be visible in any representation at all if we observe it here, and, the closer that the relations between sounds that our particular representation defines are to being the right ones for human language, the more reasonable our interpretation of the three different properties as being reflective of economy, local symmetry, and global symmetry.

## 3. Study 1

### 3.1. Materials and methods

We used the list of the speech sounds in each of the 536 languages in the P-Base phonological database of Mielke (2008)^1^. In addition to the complete list of sounds, for each language, we also extracted the lists of consonants, of stop and affricate non-sonorant consonants, and of vowels, and analyzed each separately. The feature encoding was the one that Mielke et al. (2011) adapted from Halle and Clements (1983) for use with P-Base (by adding two extra features)^2^. The feature system uses 23 features in 688 distinct combinations to code a total of 1064 sounds across all 536 languages. The fact that there are fewer distinct combinations of features used than sounds coded means that there are some sounds that have the same feature encoding. Languages that contained more than one of these sounds were considered not to be encodable in the feature system and were removed from the sample, giving a total of 438 whole inventories, 485 consonant inventories, 505 stop/affricate inventories, and 486 vowel inventories.

To generate a set of random control inventories, we used the same procedure as Mackie and Mielke (2011). We first calculated the overall frequency of all 688 feature combinations in the sample. For each of the inventories in our sample (after removing unspecifiable inventories), we sampled one random inventory of matching size, independently and with probability proportional to these overall segment frequencies. The result was a size-matched set of random control inventories. For sub-systems, we did the same, but restricted the samples to sounds attested within the given sub-system: for example, the random control sets for vowel inventories were samples containing only vowels, drawn independently with probability proportional to the overall distribution of vowels in the database.

All three of the geometric properties we measure assume that we have isolated non-redundant “dimensions of variability,” or “contrastive features” of a particular inventory. For instance, in a language with no nasal vowel and no nasal consonant, the dimension nasality does not play any role, and can be safely removed from the representation. More generally, this dimensionality reduction can be done straightforwardly, by doing a search for subspaces in which all the sounds of the inventory can be distinguished, and which are irreducible, in the sense that none of the dimensions can be removed while still keeping all the sounds in the inventory distinct. This assumption impacts what we are measuring. In the case of economy, there is no alternative: economy is a measure of how efficiently used the dimensions of variability are; without a dimensionality reduction, economy and inventory size would be the same thing. For more discussion of the effects of this choice on the other two measures, and for discussion of the relation between these measures and the notion of contrastive hierarchy (Hall, 2007; Dresher, 2009; Mackenzie, 2013), see the Supplementary Materials.

There is usually more than one irreducible (non-redundant or contrastive) set of dimensions for a given inventory (Dresher, 2009). For example, the full set of English vowels, and, in particular, the contrast between [i] and [u], can be specified either using tongue constriction backness or using lip rounding. Backness and rounding are redundant in English, and so both are not necessary; but they put the vowel sounds in different relations with one another, because the vowel sound in *lot* has back tongue constriction, like [u], but, for speakers of American English, non-rounded lips, like [i]. The encodings in different irreducible sets of dimensions are what we call *variant representations* of the same inventory. In general, the irreducible sets of dimensions may not all be of the same size for a given inventory, and give the inventory slightly different geometries.

We perform each of our analyses over a very large (often complete) sample of variant representations. We do this, rather than defining statistics for local and global symmetry based on the whole set of features, primarily because typological databases are noisy: the database that we use is based on transcriptions made by linguists. There is no guarantee that a vowel sound labeled [o] in one language is not actually phonetically the same as what may have been labeled [u] in another, nor that, for example, [u] will really refer to the same sound across the whole database. By entertaining various hypotheses about what dimensions are irrelevant to the geometry, we allow for different possible intentions on the part of the transcriber. We discuss how we deal with this more below. We discuss the level of variance between representations more in the Supplementary Materials.

In defining statistics to measure economy, local symmetry, and global symmetry, we consider one major criterion they should satisfy, which is that the measurements not be inherently correlated with each other, or with other obvious properties of the inventory (like its size). For example, as much as possible, the range of values that each one can take on should not differ as a function of the others. The approach that we take is to define the statistics sequentially: the economy measure (largely) factors out size, the local symmetry measure factors out size and economy, and the global symmetry measure factors out size, economy, and local symmetry. This necessarily builds in an epistemic priority for economy over local symmetry, and of both over global symmetry, in cases where they would otherwise be correlated. There is no problem with this, keeping in mind that the statistics are purely descriptive and not tied to any specific hypothesis about causal mechanisms.

We define our economy statistic, **Econ**, for an inventory, according to the median number of dimensions, *p*, needed to distinguish all the sounds in that inventory (the median size of any irreducible set of dimensions). If there are *s* sounds in an inventory, then **Econ** = (*s* − (*p* + 1))∕(2^*p*^ − (*p* + 1)). The denominator is the number of possible sounds that could be specified with dimension *p*, since we restrict our study to binary representations, minus the smallest possible number of sounds that could have *p* irreducible dimensions. (An inventory with *n* sounds specified using *n* or more features would necessarily have at least one of those features as redundant.) This is easy to understand intuitively: it is the number of occupied nodes in a hypercube, with a correction to ensure that the minimum is always zero, regardless of size. [**Econ** is very close to a measure called Exploitation by Hall (2007), which was also studied by Mackie and Mielke (2011), except that, first, Exploitation lacks the correction for the smallest number of possible sounds and so its minimum is not zero, and, under Mackie and Mielke's definition, it is also defined using the minimum number of features rather than the median]. The maximum and the scale do change as a function of the size of the inventory, and it is for this reason that we ensure that, when we examine a set of inventories, we compare it to a random baseline that matches their distribution of sizes (see below). See the Supplementary Materials for discussion, and for a comparison of **Econ** to other measures of economy that have been used in the literature.

We compute our local symmetry statistic, **Loc**, with respect to a particular variant representation of an inventory, by calculating the number of pairs of sounds that differ only on one dimension in that representation [$N_{mp}=\underset{i,j}{\sum }{𝟙}_{d\left(p_{i},p_{j}\right)\;=\;1}$, where *p*_*i*_ is the *i*th segment, 𝟙 is the indicator function, and *d* the distance between two segments: $d\left(p_{i},p_{j}\right)=\frac{1}{2}\underset{k}{\sum }|p_{i,k}-p_{j,k}|$, with *p*_*i, k*_ being the binary value, encoded as +1 or −1, of the *k*th dimension of the *i*th segment]. We then rank-normalize (discussed presently) so that 0 is the least locally symmetric possible score for an inventory of a given size and number of features, and 1 is the most locally symmetric: having a particular size and dimension restricts the set of possible values for the number of minimal pairs in complicated ways.

Rank normalization was done in the following way: we pooled together all of the variant representations of all the natural and random inventories from both **Study 1** and **Study 2** (*N* = 90, 989 distinct geometries), plus a large additional sample (*N* = 165, 863) of randomly generated, geometrically distinct inventories for each size and dimension appearing in the data for either **Study 1** or **Study 2**. We extracted the list of the distinct possible values of *N*_*mp*_ for a given size and dimension. A given *N*_*mp*_ score was converted into a rank in that list, and then normalized to be between zero and one by subtracting one from the rank and dividing by one less than the maximum rank. To have only one value for each inventory, we took the median over all variant representations of that inventory, omitting cases where only one *N*_*mp*_ score was possible (or at least attested) for the given size and dimension, in which case Loc adds no new information. (This led to the total omission from the analysis of **Loc** of 56 natural stop/affricate inventories, 60 control stop/affricate inventories, 61 natural vowel inventories, and 78 control vowel inventories, where, under no analysis was it possible to get a *N*_*mp*_ score that was not totally determined by the size and *p*.)

We compute our global symmetry statistic, **Glob**, with respect to a particular variant representation of an inventory, by summing, over all dimensions of variability, the absolute value of the difference between the number of sounds with each of the two binary values on that dimension ($N_{im}=\underset{k}{\sum }|\underset{i}{\sum }p_{i,k}|$, where *p*_*i, k*_ is the binary value, encoded as +1 or −1, of the *k*th dimension of the *i*th segment). As above, we convert this number to a rank from the table of possible geometries. Both this number and **Loc** are functions of the full set of distances between pairs of sounds, and are not independent (the sum of differences will be lower when the number of pairs with distance one is higher). We thus obtain **Glob** by converting the sum to its normalized rank among the possible values for a given size, dimension, and value of *N*_*mp*_, ranking the possible values in descending order so that higher values represent more balanced inventories. As a result, **Glob** is uncorrelated with either **Econ** or **Loc**. To have one number for each inventory, we take the median over all variant representations of that inventory, omitting cases where only one *N*_*im*_ score was attested or possible for the given size, dimension, and *N*_*mp*_-value, in which case Glob adds no new information. (This led to the total omission from the analysis of Glob of two natural consonant inventories, 77 natural stop/affricate inventories, 31 control stop/affricate inventories, 195 natural vowel inventories, and 102 control vowel inventories, where, under no analysis was it possible to get a *N*_*im*_ score that was not totally determined by the size, *p*, and *N*_*mp*_).

After rank normalization, the Pearson correlations of **Loc** with **Econ**, **Glob** with **Loc**, and **Glob** with **Econ**, are all zero, taken over all the distinct geometries used for computing the ranks.

To find a set of variant representations for an inventory, we use a different procedure than the one Mackie and Mielke (2011) used to obtain the smallest set of irreducible dimensions: that procedure only yields one variant representation, whereas we are looking in principle for all of them. We do a bottom-up search on the power set of the set of features. We reduce the (otherwise exponential) time complexity of the algorithm, yielding an approximate solution, which affects larger inventories and the uniform inventories of **Study 2** disproportionately. When the search moves to explore larger-size feature specifications, it does so by adding features to each of the subsets of features currently under consideration that are not yet sufficient to specify the inventory. We put a fixed upper limit on the number of subsets that can have a feature added and take a random subset of that size. It can happen that, in this way, we arrive at a set of features that is not irreducible, because we have skipped over one of its subsets. This is easy to check by keeping track of which subsets we have skipped and ensuring that the set is not a proper superset of any of them.

### 3.2. Results

**Figure 4:**
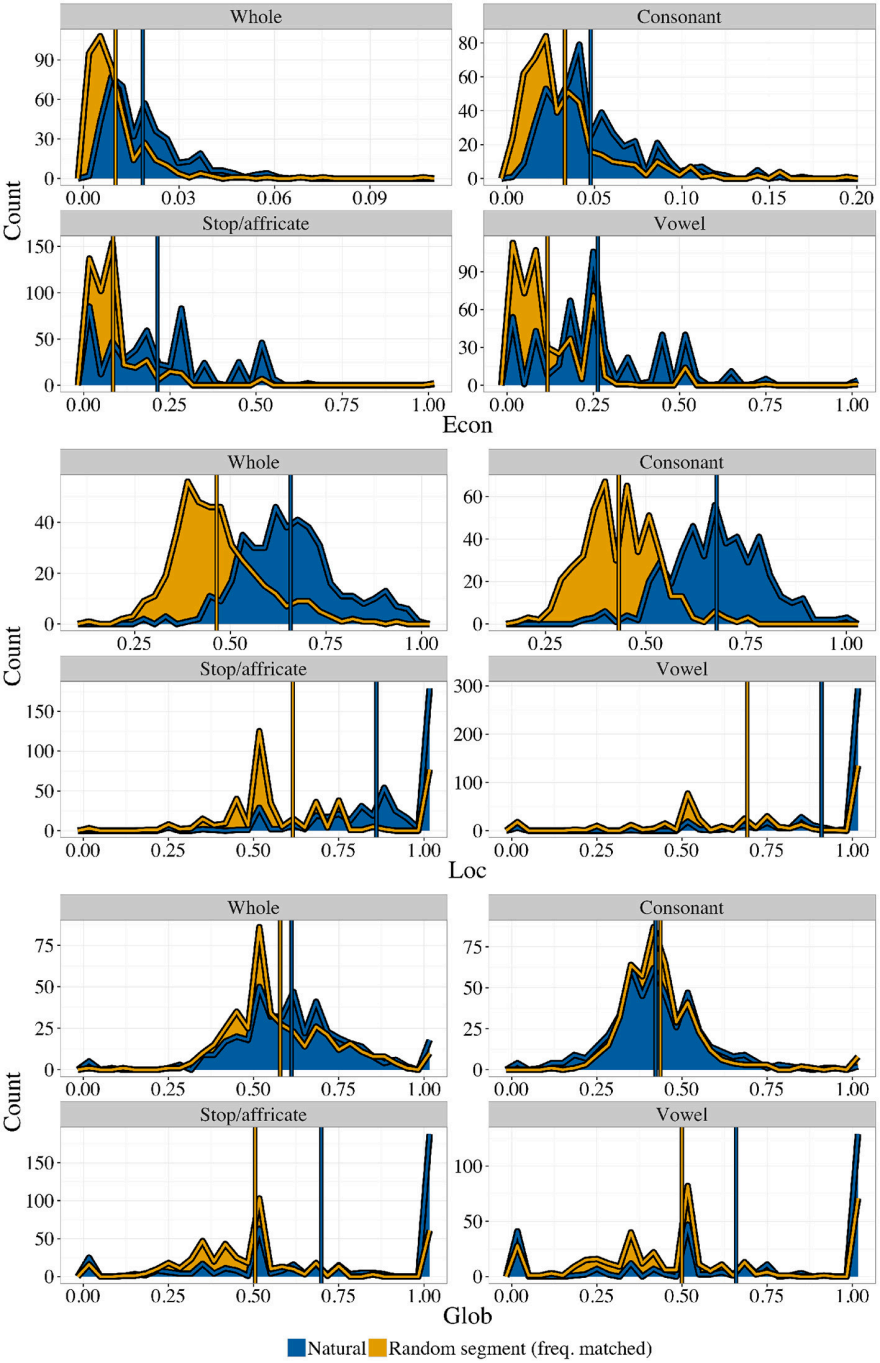
**Three geometric properties in natural vs. random inventories, taken from four defined subsets of the set of sounds in each inventory**. Blue (darker) areas are natural inventories, orange (lighter) areas are random control inventories. Both histograms are highlighted with lines of the appropriate color. Means are shown as colored vertical lines on top of the histograms. Note that, while the scales for each graph are different for readability, the theoretical minimum is always zero and the theoretical maximum is always one.

Figure 4 shows histograms and means for all three statistics, for whole inventories and for three kinds of subsystems (consonants, stops/affricates, and vowels), along with the corresponding random controls. **Econ** tends to be larger for natural inventories than would be expected if combinations of segments were independent of each other, as shown previously by Mackie and Mielke (2011). Both **Loc** and **Glob** also show higher levels in natural inventories than would be expected by chance. The size and composition of systems affects the distribution of all three statistics. Because the control inventories are frequency-matched random inventories, and are also matched for size, this is reflected in the shape of the histograms for the random control inventories as well. The set of possible values for each statistic is restricted by the fact that all three measures are discrete functions of the set of possible distinct geometries. Since the set of possible geometries grows as a function of inventory size, the smaller inventories (stop/affricate and vowel inventories) give histograms that are less smooth for all three measures.

**Figure 5:**
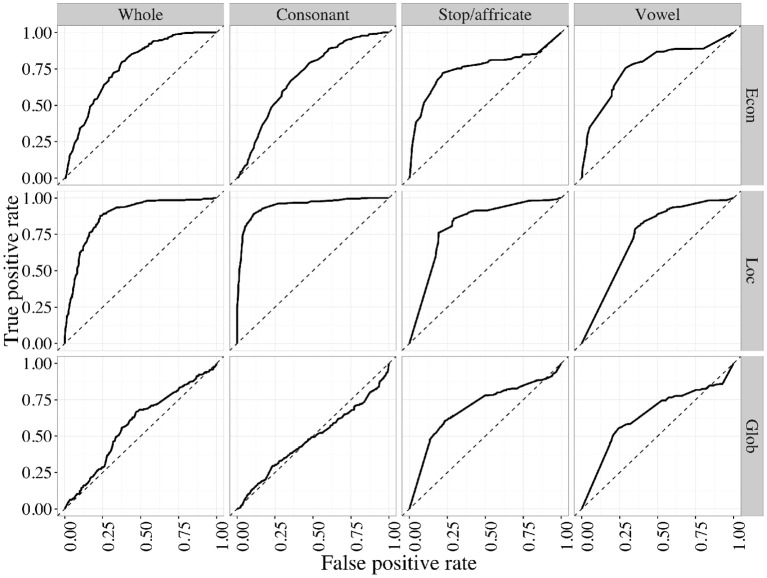
**Receiver operating characteristics for natural vs. random inventories, using each of the three geometric properties as decision criteria, within each of the four kinds of inventory subsets**.

Due to the highly non-normal character of these distributions, we use receiver operating characteristic (ROC) curves to quantify the degree of separation of the natural vs. random histograms. One can consider the histogram comparison problem as a task of deciding whether an inventory is natural or not, solely on the basis of one of the geometric properties (for example, **Econ**). If the histogram for natural inventories systematically has more area at higher levels of **Econ**, this will be possible, at least to some degree. The receiver operating characteristic (correct guesses as a function of false positives) will, on average, be a curve falling above the line *y* = *x*. Figure 5 shows ROC curves for the four subsystem types, for all three statistics.

The maximum possible area under an ROC curve (area under the curve, or AUC) is 1. Subsystem types where natural inventories have greater **Econ** will have an area >0.5, and, the larger this tendency (the more that the dark blue histogram is non-overlapping with, and to the right of, the light orange histogram), the closer the AUC will be to 1. We generate bootstrap confidence intervals on the AUC and report them in Table 1, alongside means for the four subsystems, for all three statistics. Table 1 confirms what Figure 4 shows visually: natural inventories differ systematically from a control generated at random by sampling segments independently, by having higher levels of all three of these geometric properties, with the exception of the value of **Glob** in consonant inventories (in the Supplementary Materials we also give a different analysis that takes a slightly more conservative approach to dealing with variant representations, where the result is no longer robust at the level of whole inventories; no other results change). Because the random controls are matched to natural inventories, this result demonstrates that natural inventories have different geometric properties than would be expected if sounds combined fully independently.

**Table 1:** **Mean values of Econ, Loc, and Glob, with bootstrap AUC 95% intervals for comparisons of distributions**.

	**Whole**	**Consonant**	**Stop/Affricate**	**Vowel**
**STUDY 1**				
Natural **Econ**	0.02	0.05	0.21	0.26
Random segment (freq. matched) **Econ**	0.01	0.03	0.09	0.12
Random segment (freq. matched) < Natural **Econ**	0.74–0.80	0.67–0.73	0.72–0.78	0.73–0.79
Natural **Loc**	0.66	0.68	0.86	0.91
Random segment (freq. matched) **Loc**	0.46	0.43	0.62	0.69
Random segment (freq. matched) < Natural **Loc**	0.85–0.90	0.93–0.96	0.78–0.84	0.70–0.77
Natural **Glob**	0.61	0.42	0.70	0.66
Random segment (freq. matched) **Glob**	0.58	0.44	0.50	0.50
Random segment (freq. matched) < Natural **Glob**	0.54–0.62	(0.44–0.52)	0.66–0.73	0.60–0.69
**STUDY 2**				
Random segment **Econ**	0.02	0.03	0.07	0.11
Random feature (freq. matched) **Econ**	0.03	0.04	0.10	0.13
Random feature **Econ**	0.12	0.13	0.11	0.11
Random segment: Uniform < freq. matched **Econ**	*0.21–0.27*	(0.45–0.53)	0.52–0.59	(0.48–0.55)
Random feature: Uniform < freq. matched **Econ**	*0.00–0.02*	*0.02–0.04*	*0.33–0.40*	(0.48–0.55)
Random segment < random feature **Econ**	1.00–1.00	0.99–1.00	0.71–0.77	0.52–0.59
Random segment **Loc**	0.34	0.38	0.56	0.63
Random feature (freq. matched) **Loc**	0.33	0.38	0.53	0.60
Random feature **Loc**	0.27	0.28	0.38	0.45
Random segment: Uniform < freq. matched **Loc**	0.82–0.87	0.67–0.73	0.57–0.64	0.54–0.62
Random feature: Uniform < freq. matched **Loc**	0.81–0.87	0.88–0.92	0.71–0.78	0.70–0.76
Random segment < random feature **Loc**	*0.10–0.15*	*0.08–0.11*	*0.21–0.27*	*0.26–0.32*
Random segment **Glob**	0.49	0.36	0.47	0.47
Random feature (freq. matched) **Glob**	0.30	0.31	0.46	0.47
Random feature **Glob**	0.58	0.56	0.57	0.60
Random segment: Uniform < freq. matched **Glob**	0.65–0.72	0.68–0.74	0.54–0.61	0.50–0.58
Random feature: Uniform < freq. matched **Glob**	*0.01–0.02*	*0.01–0.03*	*0.25–0.32*	*0.29–0.36*
Random segment < random feature **Glob**	0.73–0.80	0.94–0.96	0.68–0.75	0.66–0.73

***Study 1:** natural vs. frequency-matched random segment inventories; **Study 2:** uniform vs. frequency-matched random segment inventories, uniform vs. frequency-matched random feature inventories, and uniform random segment vs. uniform random feature inventories. AUC intervals above 0.5 indicate that the inventory group on the right side of the < has systematically larger values for the given geometry statistic, and AUC intervals below 0.5 (in italics) indicate that the inventory group on the left side of the < actually has systematically larger values for the given geometry statistic. Cases where the interval includes 0.5 are in parentheses*.

Global symmetry bears some conceptual similarity to the notion of dispersion, which can be operationalized as the sum of all the distances between pairs of sounds. Although they are not the same, it is likely that dispersion and global symmetry are correlated, as higher values of global symmetry imply greater distances between at least some pairs of sounds. We leave it to further work to see whether independent tendencies for global symmetry and dispersion are present in natural language inventories would remain after dispersion is factored in.

Clements (2003, 2009) claimed that there was no typological tendency for symmetry at a global level in consonant inventories. The basis for this claim was the apparent non-existence of the following, supposedly symmetrical inventory. Clements attempted to explain the non-existence of this inventory by appealing to the fact that the inventory is less than fully economical, lacking a series of voiced fricatives.

**Table:** 

Voiceless stops	p	t	k
Voiced stops	b	d	g
Voiceless fricatives	f	s	x

Intuitively, this inventory as laid out visually does demonstrate symmetry: if divided down the middle on either of the two axes, the table would have the same number of segments, in the same positions, on either side. However, this inventory is not in any coherent sense symmetrical. The argument conflates two things: the dimensions that have some causal relevance in shaping the typology of inventories, and the geometries of inventories with respect to those dimensions. Such an inventory would only be symmetrical if we believe that there is a causally relevant continuum along which voiceless stops, voiced stops, and voiceless fricatives can be ordered, with voiceless stops and voiceless fricatives being at the two end-points. If that is true, then the inventory is both symmetrical and fully economical. The inventory is not symmetrical if voicing and stop/fricative are two independent dimensions, because there are only half as many voiced as voiceless consonants, and only half as many fricatives as stops. If that is the case, then the inventory is neither fully economical nor fully symmetrical (and similarly if there is no three-place continuum for place of articulation). Here we properly evaluate global symmetry by evaluating it in the same feature space as economy, and find that, although it is indeed lacking in consonant inventories, global symmetry is high elsewhere across languages.

## 4. Study 2

**Study 2** looks at the relation between geometric properties of inventories and the frequency distribution of individual sounds or feature values in the world's languages. Some of the most frequent or least frequent sounds in the world's languages may have certain properties which influence the geometries of inventories in which those sounds occur. Conversely, the geometric properties of sound inventories may have an impact on the frequency distribution of sounds.

The random control inventories from **Study 1** were made up of real segments and were constructed to match the natural relative frequency of segments. Here we carry out a 2 × 2 manipulation of random inventories. We build inventories made up of *random features* in addition to random segments, by sampling each feature value of each sound in the inventory independently, without regard for whether the result is a real segment. Then, for both random segment inventories and random feature inventories, we manipulate whether the segments or feature values are drawn from a uniform distribution or from the empirical frequency distribution. Thus, in addition to the frequency-matched random segment inventories from **Study 1**, we now add three more kinds of random inventories: *random segment* inventories which are sampled using a uniform distribution over segments; *random feature* inventories in which each feature value ([+] or [−]) is equiprobable; and *frequency-matched random feature* inventories, in which the probability of [+] or [−] for a given feature follows the empirical probability in the database. For example, nasal vowels make up 9.8% of the world's vowel sounds, while oral vowel sounds make up the rest, so, when constructing the frequency-matched random feature inventories for vowels, the nasal dimension is sampled with a biased coin flip, with probability 0.098 of drawing a nasal sound. These frequencies were taken from the raw inventories and not from the reduced (variant) representations^3^. In the uniform random feature inventories, the probability of [+] and [−] is 0.5.

These random inventories can be seen as being closer and closer approximations to natural inventories. The frequency-matched random segment inventories are the most like natural inventories, the uniform random segment inventories are somewhat less like natural inventories, the frequency-matched random feature inventories are intuitively even less natural, because they contain many non-existent feature combinations, and the uniform random feature inventories are the least natural.

### 4.1. Materials and methods

Materials and methods were as in **Study 1**. Uniform random segment inventories were sampled just like the frequency-matched random segment inventories, except that the probability for any attested segment to be included in an inventory was equal. Random feature inventories were generated by sampling values for each feature independently. In the frequency-matched ones, the probability of one or the other of the two feature values for the first sound in the inventory was proportional to the relative frequency of that value in the whole set of inventories. Again, different probabilities were used when generating inventories to be compared against different sub-systems: for example, the probability of nasal vs. non-nasal sounds was different for vowels than for stops. Subsequent sounds were generated in the same way, except that sounds that were exactly the same as any previously sampled sound in the inventory were rejected and resampled. The distribution of inventory sizes was respected as before. As before, there are inventories for which the value of **Loc** is determined by size and *p*, and these are removed from consideration for **Loc**. For the uniform random segment inventories, 57 stop/affricate inventories and 59 vowel inventories were removed for this reason; for the uniform random feature inventories, 55 stop/affricate inventories and 52 vowel inventories; and for the frequency-matched random feature inventories, 59 stop/affricate inventories and 66 vowel inventories. Similarly, there are inventories for which the value of **Glob** is totally determined by size, *p*, and *N*_*mp*_, which are removed from consideration for **Glob**. For the uniform random segment inventories, 23 stop/affricate inventories and 76 vowel inventories were removed for this reason; for the uniform random feature inventories, 21 stop/affricate inventories and 40 vowel inventories; and for the frequency-matched random feature inventories, 32 stop/affricate inventories and 83 vowel inventories.

### 4.2. Results

**Figure 6:**
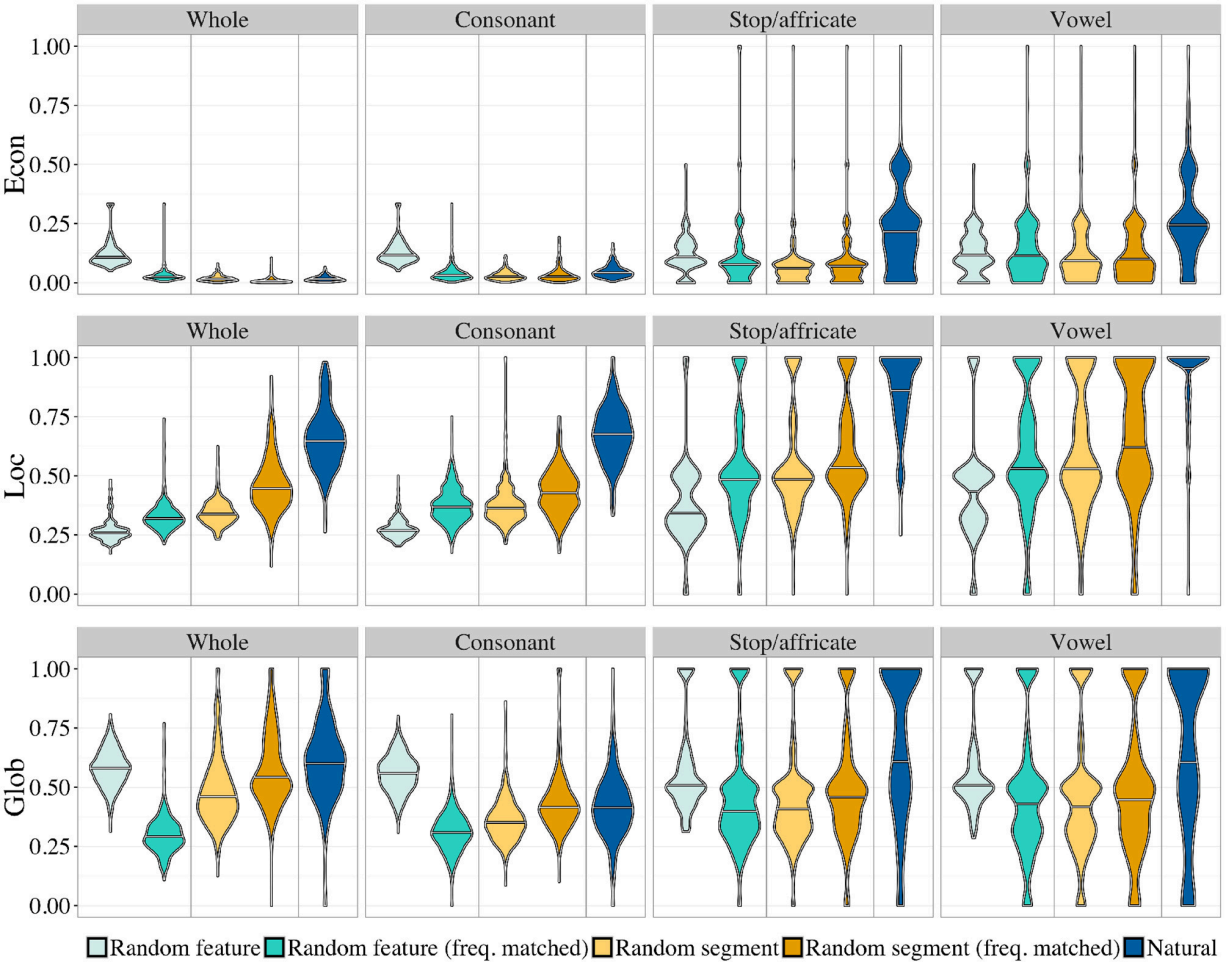
**Smoothed density plots for the three geometric properties, comparing different random inventories (see text)**. The horizontal line is the smoothed median. Control (dark blue) and frequency-matched random segment (darker orange) inventories are the same as in **Study 1**.

Figure 6 shows the distributions of the three geometry statistics for the different types of random inventories. Table 1 lists the median values and the same AUC analysis as in **Study 1** for several comparisons between different kinds of random inventories.

The pattern for **Loc** is the simplest. First, the natural inventories consistently have the highest local symmetry compared to all the random inventories. This is interpretable as a strong pressure for local symmetry, which cannot be attributed to simple statistical properties that could emerge by chance. Second, there seems to be a gradient effect within random inventories, whereby the most unnatural inventories have the lowest value of **Loc**. This can be explained by two separate factors. For the random feature inventories, the introduction of frequency biased feature values has the effect of making segments tend toward a particular corner of the hypercube, therefore increasing the local density, and therefore the number of oppositions. For the random segment inventories, in contrast, such an explanation does not work. There is no a priori reason why frequent segments should form systems that are more locally symmetric than less frequent ones. This effect has therefore to derive from properties of natural sound systems: as a matter of fact, frequent sounds tend to cluster in locally symmetric systems. This reinforces the conclusion that there is a pressure for local symmetry in natural inventories.

Next, let us examine the patterns for **Glob**. First, in most cases, natural inventories are more globally symmetric than random ones, although the effect is weaker than for local symmetry. In fact, for consonant systems, a completely unnatural system (random feature matrices) is more globally symmetric than natural inventories. Second, for random inventories, there is no gradient effect, but an interesting interaction between inventory type and frequency. For random feature inventories, a frequency bias makes inventories less globally symmetric compared to uniform. This can be explained by the same argument as above: if sounds are restricted to a corner in the hypercube because of biased feature values, the outcome will be less well-balanced than for uniformly random features. Notice, though, that this is not because the two measures, **Glob** and **Loc** are correlated: they are not. Even though, intuitively, systems with more oppositions will be less symmetrical overall, the construction of **Glob** ensures that it only measures the residual global symmetry that is not determined by **Loc**. For random segment inventories, the effect of frequency increases global symmetry. As above, this has to be attributed to properties of natural sound systems: frequent sounds tend to cluster in systems that are more globally symmetric than infrequent ones.

The most complicated pattern is that of **Econ**. For **Econ**, the pattern of results differs between “broad” inventories (whole inventories and consonant inventories), and “narrow” ones (stop and vowel inventories), which are restricted to a more coherent phonetic class (based on manner of articulation). For broad classes, natural inventories and all the random inventories except the random feature inventories have a very low level of economy (the mean **Econ** is below 0.05). For narrow classes, in contrast, these inventories are all relatively more economical. The drop in economy for broad inventories is likely attributable to something very general: a very diverse pool of sounds will not yield very economical inventories compared to a highly specialized pool of sounds, because it mixes different classes of segments that require different articulators, and therefore cannot share too many features. The source pool of segments used in the random stop/affricate and vowel inventories is subject to a narrow, hard restriction (in the random segment inventories), or a narrow but soft restriction to stop-like or vowel-like sounds (in the frequency-matched random feature inventories). A similar restriction to consonants is evidently too broad: here we no longer see high levels of economy. There is no restriction at all on the pool of segments in the uniform random feature inventories. In the uniform random feature inventories, the only thing that differs between the four different cases—whole, consonant, stop/affricate, vowel—is the distribution of inventory sizes, which matches the natural one in each case. **Econ** is not completely independent of inventory size (see Supplementary Materials), but this does not change the conclusion: there is an interaction between broad vs. narrow, on the one hand, and uniform feature vs. the rest, on the other. Inventory size drives part of the difference between broad and narrow inventories, but not all, because it affects uniform feature inventories differently from the others. (In fact, it can be seen that the effect of size is negligible for the random feature inventories.) Unlike for **Loc** and **Glob**, segment frequency does not have a consistent effect on **Econ**: it is not the case that more frequent sounds cluster together into a set that is particularly economical.

The results for **Econ** are partly consistent with the claims of Clements (2009) about the relation between markedness (for our purposes, any non-uniform frequency distribution, either of segments or feature values) and economy. Clements observes that markedness will reduce economy by restricting the pool of available segments that an inventory can contain, thus pushing the geometry away from the ideal 2^*k*^ segments on *k* features. As we note, this is true for broad inventories, and it is also true in general for **Glob**.

## 5. Discussion

This paper confirms, with a different analysis, a previously documented tendency for natural sound inventories to be economical (a small number of feature dimensions account for the differences between sounds in a single inventory). In addition, based on our geometric interpretation of sound systems, we demonstrated two kinds of pressures toward symmetry, one local and one global. These tendencies are found at the level of sub-inventories (like the inventories of consonants, stops/affricates, or vowels) as well as for entire inventories. They are present above and beyond the pressure that may arise from the fact that certain sounds or certain feature values are more frequent than others across languages. In addition to this, the crosslinguistically most frequent sounds form a set that is significantly more coherent in terms of local and global symmetry than the whole pool of attested sounds. This is not trivial and the explanation is still unknown; one possible reason is that frequency distribution of sounds across languages has itself been influenced by tendencies in inventories, with the frequency of sounds that stand in symmetrical relations with high frequency sounds being given a boost.

The causal mechanism behind these tendencies is unknown, but it must be one that constrains language change in some way. An inventory is the outcome of a dynamic process in which languages are re-transmitted over historical time. When two sounds merge into one, or when a sound shifts its pronunciation, or when a sound splits into two sounds, over the history of a language, there is at least some tiny pressure to maintain relatively high levels of **Econ**, **Glob**, and **Loc**. The scientific question is what kind of dynamical system would tend to give the kinds of geometries we see in natural sound inventories. Part of the explanation may be cognitive. Some biases introduced by the cognitive apparatus for learning language could even embody these pressures in an obvious way. For example, in learning the motor aspect of language production, the brain of the infant might be trying to minimize the number of controllable degrees of freedom. After having successfully mastered the articulation of a contrast in voicing for [p] vs. [b], the production system would preferentially reuse this contrast for another class of segments, such as fricatives, leading to greater **Econ**, and possibly greater **Loc**, depending on the precise nature of the pressure (see Lindblom, 2010).

On the other hand, systematic pressures might exist that have come from non-cognitively-controllable aspects of the speech signal, such as the mechanics of the vocal apparatus or the spectral characteristics of common environmental noise. “Channel bias” might exist in the form of systematicies in how the speech signal is distorted during transmission by these external forces.

In fact, both cognitive and non-cognitive forces likely have an impact on the geometries of sound inventories. For example, modern East Slavic languages (Russian, Ukrainian, Belarusian, Rusyn) have two parallel series of consonants, one palatalized (“soft”: [k^j^], [d^j^], [s^j^], and so on), and another not (“hard”: [k], [d], [s], and so on). Which non-palatalized consonants have palatalized counterparts depends on the language, but all these languages have a large number of consonant oppositions related by the same palatalized/non-palatalized feature distinction [a geometric situation Trubetzkoy (1939) referred to as “correlation”], with the result of high local and global symmetry. This was not the case in Early Common Slavic, where the palatalized consonants were just contextual variants of their non-palatalized counterparts, before front vowels. When some front vowels stopped being pronounced, the palatalized versions could stand on their own, becoming members of the inventory of Late Common Slavic apparently all at once (Jakobson, 1929).

The existence of a large set of consonants with palatalized contextual variants was a prerequisite to this change in the inventory of Common Slavic. Cognitive factors certainly contributed to this. Distinguishing two variant pronunciations consistently according to context is something that speakers of a language learn and re-learn at each generation, as evidenced by the fact that not all languages have the same contextual variation (even dialects of the same language can vary in very fine phonetic details of this kind: Tamminga and Zellou, 2015). Appropriate cognitive apparatus is therefore required for this pattern to be reproduced over generations. The nature of the perceptual apparatus presumably also contributes via the fact that perceptual cues for front vowels are somewhat ambiguous as to whether they were generated by a front vowel or by a palatalized consonant (Hamann, 2003). But biomechanics plays a clear role in allowing the contextual palatalization to be so widespread in the first place: any consonant produced with the tongue will require the tongue to make a transition to a palatal articulation when it is produced before a front vowel; the articulation will be under pressure to advance toward a palatal place of articulation over time. This will be true for a large set of consonants. Non-cognitive aspects of the speech channel, such as the structure of the vocal apparatus itself, could therefore be causally relevant for inventory geometries.

We leave a thorough exploration of these questions, both experimental and through assessment of models of language change and language acquisition in terms of their predicted geometries, for future research. One model that was used to simulate the dynamics of language change for which geometry has been partly measured is that of De Boer (2000), which simulates a dynamic process of language change in vowel inventories using a vowel formant synthesis model. In this model, vowel categories merge over time in a way that could be interpreted as reflecting a mix of the limits of the (simulated) perceivers and the limits of the (simulated) acoustic medium. Meanwhile, new vowel categories are sometimes formed close to the ones that the agents in the simulation hear—with their locations determined in a way that could be interpreted as capturing both cognitive influences and the limits of the acoustic medium—while other new categories appear randomly throughout the space. Clearly these details of the dynamical system will affect the resulting geometries.

A set of 29 artificial vowel inventories generated by this process were presented by Mackie and Mielke (2011), who observed that, on several related economy measures, these inventories were similar to natural vowel inventories. In light of the results of Study 2, in which some random inventories generated by highly unnatural processes were shown to be more economical than natural ones, it is worth re-examining these inventories in order to see if this model is indeed a plausible one. The mean **Econ**, **Loc**, and **Glob** for the inventories cited are in fact far below the mean values for natural vowel inventories (**Econ**: 0.15 vs. 0.26 for natural vowel inventories; **Loc**: 0.54 vs. 0.91, natural; **Glob**: 0.26 vs. 0.66, natural). Similarly, a 95% confidence interval of AUCs generated by comparing against 10,000 randomly drawn size-matched samples of natural vowel inventories shows systematically higher values for natural inventories for all three geometric statistics (**Econ**: 0.56–0.72, **Loc**: 0.71–0.83, **Glob**: 0.64–0.89)^4^. We therefore take the question of what kind of dynamic process could yield natural inventory geometries to be entirely open.

Whatever the explanation, the three geometric pressures we demonstrate cannot be the only ones governing sound change. Otherwise, all languages would end up in the absolute maxima of **Econ**, **Glob**, and **Loc**. Such systems would correspond to completely saturated hypercube geometries, where all the nodes are occupied by a segment (**Econ** = 1), and both local and the global symmetry are at their relative maxima (**Loc** = 1, **Glob** = 1). Counteracting pressures could come from a tendency to maintain contrast at the lexical level. For example, in an already dense inventory, all possible shifts and splits of sounds might yield already existing sounds. Maintaining contrast would leave as the only possible change a non-economical one, which adds a new dimension.

One piece of evidence suggesting that there are broad structural factors that affect inventories that may counteract economy, local symmetry, and global symmetry is a shift in pronunciation that is currently happening independently in dialects of English all over the world: the tongue constriction of the [u] sound in *goose* is shifting toward the position of the [i] sound at the front of the mouth [see Hall-Lew (2011) for a summary]. The convergent development of *goose-fronting* across distantly related dialects suggests that something about the common structure of the inventory of English encourages such a change. But *goose*-fronting is paradoxical from the point of view of economy, because its end state (arguably already been reached in some British dialects: Harrington et al., 2011) is one in which *goose* is pronounced with the [y] sound, which introduces an opposition with [i] based crucially on lip rounding into the English vowel inventory. No such oppositions have been present in English since before the time of Chaucer, and adding one runs in the face of economy. It also weakens local symmetry by moving [u] away from its immediate phonetic neighbors in *foot* and *goat*, and creates imbalance between vowels with tongue body constriction in the back vs. the front of the mouth, at least in dialects where the sounds in *lot* and *thought* have merged. Labov (1994) suggests that there is simply a universal tendency toward fronting of back vowels. Alternatively, a pressure toward maximal dispersion of sounds throughout the space, if it were as we have measured geometric properties here, with respect to a reduced set of contrastive dimensions, would actually serve as a counter-pressure to economy. The dispersion of an inventory goes up as a function of the total of the distances between all pairs of sounds. If the distance between the sounds were suddenly computed with an additional dimension included (as it would be if an additional dimension was forced into being contrastive by the presence of an additional sound), then the distances would all increase (or at least not decrease), thus increasing the dispersion. It is therefore worth attempting to measure other kinds of non-independence between sounds in large scale typological studies.

## Author contributions

The first author performed the analysis. Both authors contributed to the conception of the analysis and wrote the paper.

## Funding

Research was funded by the European Research Council (ERC-2011-AdG 295810 BOOTPHON), the Agence Nationale pour la Recherche (ANR-2010-BLAN-1901-1 BOOTLANG), and the Fondation de France. It was also supported by ANR-10-IDEX-0001-02 PSL and ANR-10-LABX-0087 IEC.

## Availability of sources

All data and analysis scripts used in this paper can be retrieved at http://github.com/bootphon/contrastive-symmetry/.

### Conflict of interest statement

The authors declare that the research was conducted in the absence of any commercial or financial relationships that could be construed as a potential conflict of interest.

## Footnotes

^1^Inventories and code for annotating them in this feature system were those of Mielke (2008). Mackie and Mielke (2011) used the same database, but the total number of inventories given there is 549. That is because the database also includes multiple varieties (dialects) for some languages (most of which all have the same inventory, but a few of which are slightly different); a total of 628 varieties are present. Only one, standard variety is chosen as the representative of each language, but what counts as a single language depends on how varieties are grouped. The version of P-Base we had access to grouped varieties into languages according to a slightly newer standard (ISO-639-3 codes), and so included a slightly different set of inventories than Mackie and Mielke (2011).

^2^The feature encoding is superficially ternary (some features are specified as 0, rather than + or −, for some sounds). This is only superficial, as no set of three sounds is crucially distinguished by three values of the same feature. In most inventories, the 0 values of most features do not crucially distinguish any individual pairs of sounds, and in these cases we treated 0 as −. In cases where a 0 value for one feature was all that distinguished one sound from another, we treated 0 as the appropriate contrasting value, either − or +.

^3^But they were computed on the modified representation with only + and − described in footnote 2.

^4^Our measure for economy is conceptually different from any of Mackie and Mielke's in that it uses a median, rather than a minimum, number of features, which leads us to somewhat more conservative numbers for a given inventory. Replacing **Econ** with a version that uses the minimum possible dimension of any analysis, the artificial vowel inventories are indeed indistinguishable from natural ones: although the mean value of the statistic is somewhat lower (0.22 vs. 0.28 for natural vowel inventories), a 95% confidence interval of AUC scores generated by comparing against 10,000 randomly drawn size-matched samples of the natural vowel inventories includes 0.5 (0.47–0.67). That could suggest that the artificial inventories are drawn from the same geometry distribution as natural ones, but the other measures presented in the text demonstrate that this is an artifact.
