# Supplementary material for: Geometric Constraints on Human Speech Sound Inventories
Source: Front Psychol. 2016 Jul 12;7:1061. doi: 10.3389/fpsyg.2016.01061 (PMC4940385; doi:10.3389/fpsyg.2016.01061)
Supplement: Supplementary file 1 [file DataSheet1.pdf]

frontiers

Supplementary Material: Geometric constraints on human speech sounds

Ewan Dunbar* **and Emmanuel Dupoux**
Laboratoire de Sciences Cognitives et Psycholinguistique (ENS–EHESS–CNRS), Département des Études Cognitives, École Normale Supérieure–PSL Research University, Paris, France

Correspondence*:
Ewan Dunbar
Laboratoire de Sciences Cognitives et Psycholinguistique (ENS–EHESS–CNRS), Département des Études Cognitives, École Normale Supérieure, Pavillon jardin, 29 rue d'Ulm, Paris 75005, France, emd@umd.edu

1 PROPERTIES OF THE THREE GEOMETRIC MEASURES

As noted in the main text, **Econ** is similar to Exploitation (Hall 2007), except that Exploitation lacks the correction for the smallest number of possible sounds and so its minimum is not zero. Measures like **Econ** and Exploitation are strongly preferable to the popular measure s/p, simply called Economy in Clements 2003. That statistic does not align with an intuitive notion of economy when comparing sets of different dimensions: an inventory on three dimensions of size eight, the largest number of sounds that can be specified on three binary dimensions, is scored 2.67, while an inventory on five dimensions of size fourteen, less than half of the possible sounds on five binary dimensions, scores higher, with 2.8. **Econ** corrects this.

Since **Loc** and **Glob** are based on normalized ranks conditional on inventory size, their range is the same (between zero and one) regardless of inventory size. For **Econ**, the theoretical range is limited: between 2^k and 2^{k+1} sounds, a perfect score for **Econ** is impossible. The upper limit increases as the number of sounds gets closer to 2^{k+1}. Since these gaps become larger on a logarithmic scale, larger inventory sizes are a priori more likely to have their **Econ** values capped in this way. The scale, which is unevenly spaced between zero and one to begin with, is also different depending on inventory size. Most of the possible values are close to zero. This is shown in Figure 1.

In principle it would be possible to totally remove any effect of inventory size from **Econ** using a normalized rank, as we do for **Loc** and **Glob**. We have not taken this step. Most of our crucial comparisons are between size-matched sets of inventories anyway, so that differences in the distribution of **Econ** across comparison sets must be due to their composition. The only case where this is not true is in the explicit comparisons between whole inventories, consonant inventories, stop/affricate inventories, and vowel inventories, where the distribution of sizes differs, but, in Study 2, we only look at an interaction between broad/narrow inventory (confounded with size) and uniform feature/other.

Furthermore, as noted in Study 2, the uniform random feature inventories, which differ only in their size distribution and not in their composition across the four classes, do not show substantial differences in central tendency for **Econ**. The practical significance of the sensitivity of the scale to inventory size is mitigated in these sets, compared to what the impact would be if **Econ** were equally likely to take on any of its logically possible values for a given size. We see this in Figure 2, which compares the median and mean **Econ** in uniform random feature inventories from Study 2 (all pooled together) to those in sample of **Econ** values, matching the distribution of sizes, in which some number of features is simply drawn uniformly at random from the range of possible values for a given size, and **Econ** is computed from that. Intuitively,

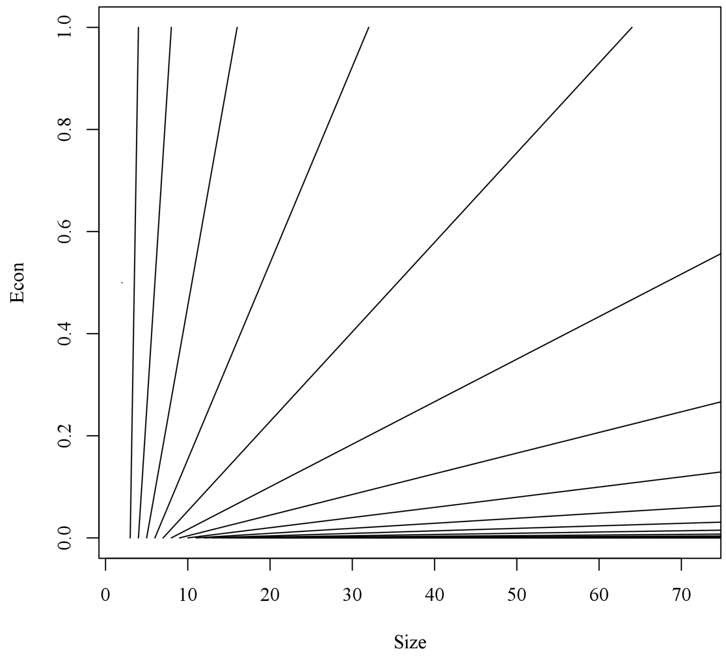

Figure 1. Logically possible values of **Econ** versus inventory size.

these are hypothetical inventories that are equally likely to fall on any of the lines in Figure 1. Natural inventories are given for reference. There is always instability for small inventory sizes, after which the random **Econ** values see their mean fall off as a function of size, and their median sharply so. For random inventories, however, this pattern is attenuated; **Econ** falls off a bit, but remains relatively stable. These inventories actually tend to have relatively large levels of **Econ**, and thus the dramatic shrinking of the scale for the smaller values of **Econ** does not always pose a problem. For the natural inventories, this is more of an issue, and, across the board, the limitation of full economy to powers of two will clearly limit our ability to compare inventories of different sizes.

2 IMPACT OF USING CONTRASTIVE FEATURES ON LOC AND GLOB

All three measures assume that that only the irreducible dimensions ("contrastive features") are relevant to the geometry. In the case of **Loc**, adding in non-contrastive features would change the matrix of distances (hence, the geometry). For natural segments there is a fair amount of redundancy in the feature representation we use, so this would substantially reduce the number of pairs with distance equal to one. A measure of something similar could be constructed by replacing all the distances between pairs of sounds with their rank, and defining oppositions to be pairs with minimum distance (whatever that is for the given inventory). In the case of **Glob**, variant representations are more relevant. The calculation of **Glob** when non-contrastive features are added would remain unchanged, except that additional terms would be added to the sum.

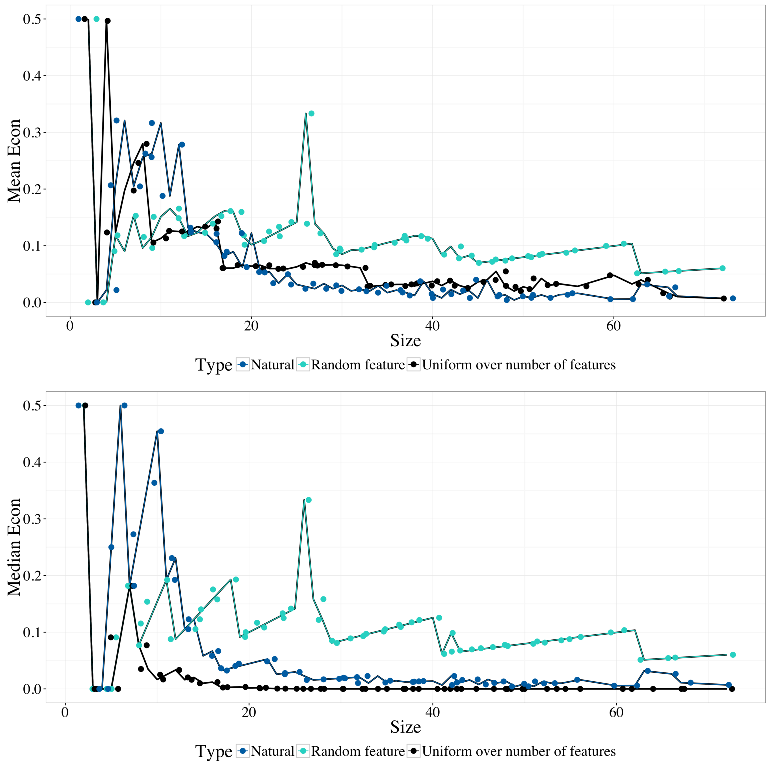

Figure 2. Mean and median economy by size in Study 2 uniform random feature inventories (turquoise) versus a size-matched sample of uniformly drawn **Econ** values (black). A small amount of jitter is added to the points to avoid overplotting.

3 RELATION TO THE CONTRASTIVE HIERARCHY

Many phonological theories not only claim that non-contrastive features have little or no impact on lexical storage in a language, but also make the further claim that there is a cognitively impactful order on features (a "contrastive hierarchy"). A contrastive hierarchy per se does not play a direct role in any of our statistics, but all three can be interpreted through the lens of a contrastive hierarchy. On a binary feature representation, a contrastive hierarchy for an inventory induces a maximum-binary branching tree where each leaf is a single sound. The pair of nodes under the root split are the sounds marked [-] and [+] respectively for the first feature in the hierarchy. If a node contains only one sound, it has no children; if it contains more than one sound which all have the same value for the next feature in the hierarchy then it has one child node, equal to the same set of sounds; otherwise, it has two children, respectively, the sounds bearing [-] and [+] for the next feature in the hierarchy.

From this perspective, **Econ** is constructed using the median of all possible tree depths, given all possible sets of contrastive features for the inventory. Each set of contrastive features yields only one tree depth, regardless of the contrastive hierarchy over those features. **Loc** and **Glob**, on the other hand, can be seen as combining information from different contrastive hierarchies for a given set of contrastive features.

Loc considers each contrastive feature in turn and imagines it at the bottom of a contrastive hierarchy. It measures how many branching nodes there would be at this bottom level of the tree. If the inventory were perfectly economical, then there would be 2^{p-1} branching nodes, where p is the number of features. It then sums all these numbers and computes a normalized rank over the possible values among trees of a given depth with a given number of leaf nodes, a step which can be seen as a way of expressing indifference to or uncertainty about the ordering of features in the hierarchy. The final step takes the median over all

analyses into contrastive features, which can be seen as a way of expressing indifference to or uncertainty about the set of contrastive features.

Glob considers each contrastive feature in turn and imagines it at the top of a contrastive hierarchy. It measures the imbalance between the number of sounds in each of the two nodes at the top level. It then sums all these numbers and computes a normalized rank over the possible values among trees of a given depth with a given number of leaf nodes, with a given number of branching nodes (which restricts the shape of the tree further in such a way as to take into account the fact that the number of branching nodes is informative about how much imbalance there could be). This step can again be seen as a way of expressing indifference to or uncertainty about the ordering of features in the hierarchy. The final step takes the median over all analyses into contrastive features, which again can be seen as a way of expressing indifference to or uncertainty about the set of contrastive features.

To actually exploit the idea of a contrastive hierarchy and incorporate it into inventory geometry, a measure would need to be constructed that considers only a single order at once and combines (different) information from the different levels of the hierarchy.

4 VARIANT REPRESENTATIONS

The number of variant representations is small for some inventories, and large for others, with a large spread (between one and 14,640). Generally speaking, the number of variant representations for larger inventories is larger, and the number can be very large for random feature inventories.

To assess the risk of taking the median—that is, the risk that this might yield unreasonably high values among the variant representations—we also performed a replication of Study 1 and Study 2 in which we drew a variant representation uniformly at random rather than taking the median. The results of this are exactly the same, qualitatively, as in the main text, except that the difference in **Glob** for whole inventories is no longer evident. The results are summarized in Table 1.

Table 1. Mean values of **Econ**, **Loc**, and **Glob**, with bootstrap AUC 95% intervals for comparisons of distributions in a replication of Study 1 and Study 2 in which a variant representation was drawn uniformly at random. The first rows are the means. AUC intervals above 0.5 indicate that the inventory group on the right side of the < has systematically larger values for the given geometry statistic, and AUC intervals below 0.5 (in italics) indicate that the inventory group on the left side of the < actually has systematically larger values for the given geometry statistic. Cases where the interval includes 0.5 are in parentheses.

	Whole	Consonant	Stop/affricate	Vowel
Natural **Econ**	0.02	0.05	0.21	0.23
Random segment (freq. matched) **Econ**	0.01	0.04	0.09	0.11
Random segment (freq. matched) < Natural **Econ**	0.66–0.73	0.64–0.71	0.79–0.85	0.80–0.87
Natural **Loc**	0.65	0.67	0.85	0.84
Random segment (freq. matched) **Loc**	0.46	0.45	0.60	0.66
Random segment (freq. matched) < Natural **Loc**	0.76–0.82	0.80–0.85	0.74–0.80	0.64–0.71
Natural **Glob**	0.58	0.43	0.62	0.66
Random segment (freq. matched) **Glob**	0.58	0.45	0.46	0.43
Random segment (freq. matched) < Natural **Glob**	(0.48–0.55)	(0.43–0.51)	0.61–0.69	0.66–0.75
Random segment **Econ**	0.02	0.04	0.08	0.11
Random feature (freq. matched) **Econ**	0.04	0.05	0.11	0.12
Random feature **Econ**	0.11	0.13	0.14	0.13
Random segment: Uniform < freq. matched **Econ**	*0.30–0.38*	(0.45–0.52)	(0.50–0.58)	(0.45–0.54)
Random feature: Uniform < freq. matched **Econ**	*0.05–0.08*	*0.07–0.10*	*0.30–0.37*	*0.40–0.49*
Random segment < random feature **Econ**	0.97–0.99	0.94–0.96	0.74–0.80	0.56–0.65
Random segment **Loc**	0.35	0.38	0.53	0.58
Random feature (freq. matched) **Loc**	0.35	0.38	0.52	0.58
Random feature **Loc**	0.26	0.28	0.39	0.46
Random segment: Uniform < freq. matched **Loc**	0.66–0.73	0.59–0.66	0.55–0.62	0.54–0.61
Random feature: Uniform < freq. matched **Loc**	0.64–0.71	0.63–0.70	0.60–0.67	0.57–0.65
Random segment < random feature **Loc**	*0.28–0.35*	*0.29–0.35*	*0.33–0.40*	*0.36–0.43*
Random segment **Glob**	0.49	0.37	0.41	0.39
Random feature (freq. matched) **Glob**	0.32	0.31	0.40	0.39
Random feature **Glob**	0.58	0.57	0.52	0.54
Random segment: Uniform < freq. matched **Glob**	0.59–0.66	0.60–0.67	0.53–0.61	0.51–0.61
Random feature: Uniform < freq. matched **Glob**	*0.09–0.14*	*0.08–0.12*	*0.31–0.39*	*0.29–0.37*
Random segment < random feature **Glob**	0.62–0.69	0.81–0.86	0.61–0.69	0.64–0.72

Supplementary Table 2. Feature representation used to generate inventory geometries (Mielke et al. 2011).

	anterior	labial	back	high	ATR	lateral	distributed	LONG	spread	low	syllabic	continuant	nasal	sonorant	coronal	tense	constr	vocalic	strident	EXTRA	consonantal	voice	round
dx, p͡k, tx, tç, t̪͡k	-	-	-	-	-	-	-	-	-	-	-	-	-	-	-	-	-	-	-	-	+	-	-
b͡g, dɟ	-	-	-	-	-	-	-	-	-	-	-	-	-	-	-	-	-	-	-	-	+	+	-
b͡tʃ, kf	-	-	-	-	-	-	-	-	-	-	-	-	-	-	-	-	-	-	+	-	+	-	-
b͡dʒ	-	-	-	-	-	-	-	-	-	-	-	-	-	-	-	-	-	-	+	-	+	+	-
ʈ, ʈr, ʈʲ	-	-	-	-	-	-	-	-	-	-	-	-	-	-	+	-	-	-	-	-	+	-	-
ɖ, ɖr, ɖʲ	-	-	-	-	-	-	-	-	-	-	-	-	-	-	+	-	-	-	-	-	+	+	-
tʂ, ʈʂ	-	-	-	-	-	-	-	-	-	-	-	-	-	-	+	-	-	-	+	-	+	-	-
dʐ, ɖʐ	-	-	-	-	-	-	-	-	-	-	-	-	-	-	+	-	-	-	+	-	+	+	-
ɖ˞	-	-	-	-	-	-	-	-	-	-	-	-	-	-	+	-	+	-	-	-	+	-	-
tʂ’, ʈʂ’	-	-	-	-	-	-	-	-	-	-	-	-	-	-	+	+	+	-	+	-	+	-	-
ɽ	-	-	-	-	-	-	-	-	-	-	-	-	-	+	+	-	-	+	-	-	+	+	-
ʈⁿ	-	-	-	-	-	-	-	-	-	-	-	-	+	-	+	-	-	-	-	-	+	-	-
ɖ, ɳɖr	-	-	-	-	-	-	-	-	-	-	-	-	+	-	+	-	-	-	-	-	+	+	-
N?, m͡ŋ	-	-	-	-	-	-	-	-	-	-	-	-	+	+	-	-	-	-	-	-	+	+	-
ɳ	-	-	-	-	-	-	-	-	-	-	-	-	+	+	+	-	-	-	-	-	+	+	-
ʂ	-	-	-	-	-	-	-	-	-	-	-	+	-	-	+	-	-	-	+	-	+	-	-
ʐ	-	-	-	-	-	-	-	-	-	-	-	+	-	-	+	-	-	-	+	-	+	+	-
e̜	-	-	-	-	-	-	-	-	-	-	-	+	-	+	-	-	-	-	-	-	-	+	-
ɻ	-	-	-	-	-	-	-	-	-	-	-	+	-	+	+	-	-	-	-	-	-	+	-
N̩	-	-	-	-	-	-	-	-	-	-	+	-	+	+	-	-	-	-	-	-	+	+	-
e̞, ɛ, ɛ̞	-	-	-	-	-	-	-	-	-	-	+	+	-	+	-	-	-	+	-	-	-	+	-
ɛʔ	-	-	-	-	-	-	-	-	-	-	+	+	-	+	-	-	+	+	-	-	-	+	-
ɛ̃	-	-	-	-	-	-	-	-	-	-	+	+	+	+	-	-	-	+	-	-	-	+	-
ɛ̃ʔ	-	-	-	-	-	-	-	-	-	-	+	+	+	+	-	-	+	+	-	-	-	+	-
ɦ	-	-	-	-	-	-	-	-	-	+	-	+	-	+	-	-	-	-	-	-	-	+	-
ɦ̃	-	-	-	-	-	-	-	-	-	+	-	+	+	+	-	-	-	-	-	-	-	+	-
æ̞	-	-	-	-	-	-	-	-	-	+	+	+	-	+	-	-	-	+	-	-	-	+	-
tçʰ	-	-	-	-	-	-	-	-	+	-	-	-	-	-	-	+	-	-	-	-	+	-	-
ʈʰ	-	-	-	-	-	-	-	-	+	-	-	-	-	-	+	+	-	-	-	-	+	-	-
ɖʰ	-	-	-	-	-	-	-	-	+	-	-	-	-	-	+	+	-	-	-	-	+	+	-
ʈʂʰ	-	-	-	-	-	-	-	-	+	-	-	-	-	-	+	+	-	-	+	-	+	-	-
ɽʰ	-	-	-	-	-	-	-	-	+	-	-	-	-	+	+	+	-	+	-	-	+	+	-
ʂʰ	-	-	-	-	-	-	-	-	+	-	-	+	-	-	+	+	-	-	+	-	+	-	-
ɛ̥	-	-	-	-	-	-	-	-	+	-	+	+	-	+	-	-	-	-	-	-	-	-	-
ʔ	-	-	-	-	-	-	-	-	+	+	-	-	-	+	-	-	+	-	-	-	-	-	-
h	-	-	-	-	-	-	-	-	+	+	-	+	-	+	-	-	-	-	-	-	-	-	-
h	-	-	-	-	-	-	-	-	+	+	-	+	-	+	-	+	-	-	-	-	-	-	-
h̃	-	-	-	-	-	-	-	-	+	+	-	+	+	+	-	-	-	-	-	-	-	-	-
h̃	-	-	-	-	-	-	-	-	+	+	-	+	+	+	-	+	-	-	-	-	-	-	-
aɪ, iɛ, œi, ɔi, ɔi̯, ɛː, ɪɛ	-	-	-	-	-	-	-	+	-	-	+	+	-	+	-	-	-	+	-	-	-	+	-
ɛ̃ː	-	-	-	-	-	-	-	+	-	-	+	+	+	+	-	-	-	+	-	-	-	+	-
t̪͡c	-	-	-	-	-	-	+	-	-	-	-	-	-	-	-	-	-	-	-	-	+	-	-
t̪ʃ	-	-	-	-	-	-	+	-	-	-	-	-	-	-	-	-	-	-	+	-	+	-	-
b̪ʒ, d̪ʒ	-	-	-	-	-	-	+	-	-	-	-	-	-	-	-	-	-	-	+	-	+	+	-
pʃ’	-	-	-	-	-	-	+	-	-	-	-	-	-	-	-	+	+	-	+	-	+	-	-
t̻ɕ	-	-	-	-	-	-	+	-	-	-	-	-	-	-	+	-	-	-	+	-	+	-	-
d̻ʑ, d̻ʒ̻	-	-	-	-	-	-	+	-	-	-	-	-	-	-	+	-	-	-	+	-	+	+	-
t̻ɕ’	-	-	-	-	-	-	+	-	-	-	-	-	-	-	+	+	+	-	+	-	+	-	-
ɲ͡n̪	-	-	-	-	-	-	+	-	-	-	-	-	+	+	-	-	-	-	-	-	+	+	-

	anterior	labial	back	high	ATR	lateral	distributed	LONG	spread	low	syllabic	continuant	nasal	sonorant	coronal	tense	constr	vocalic	strident	EXTRA	consonantal	voice	round
fʃ	-	-	-	-	-	-	+	-	-	-	-	+	-	-	-	-	-	-	+	-	+	-	-
pʃʰ	-	-	-	-	-	-	+	-	+	-	-	-	-	-	-	+	-	-	+	-	+	-	-
t̻ɕʰ	-	-	-	-	-	-	+	-	+	-	-	-	-	-	+	+	-	-	+	-	+	-	-
ɭ̊	-	-	-	-	-	+	-	-	-	-	-	-	-	+	+	-	-	+	-	-	+	+	-
e, emf, e̝, ɛ̝	-	-	-	-	+	-	-	-	-	-	+	+	-	+	-	+	-	+	-	-	-	+	-
eʔ, eʔe	-	-	-	-	+	-	-	-	-	-	+	+	-	+	-	+	+	+	-	-	-	+	-
ẽ, ẽ̝	-	-	-	-	+	-	-	-	-	-	+	+	+	+	-	+	-	+	-	-	-	+	-
ẽʔẽ	-	-	-	-	+	-	-	-	-	-	+	+	+	+	-	+	+	+	-	-	-	+	-
æ	-	-	-	-	+	-	-	-	-	+	+	+	-	+	-	+	-	+	-	-	-	+	-
æ̃	-	-	-	-	+	-	-	-	-	+	+	+	+	+	-	+	-	+	-	-	-	+	-
e̥	-	-	-	-	+	-	-	-	+	-	+	+	-	+	-	+	-	-	-	-	-	-	-
e̤	-	-	-	-	+	-	-	-	+	-	+	+	-	+	-	+	-	+	-	-	-	+	-
ẽ̤	-	-	-	-	+	-	-	-	+	-	+	+	+	+	-	+	-	+	-	-	-	+	-
ai, ai̯, ei, ej, eu, eə, eɪ, eˑ, e̝ˑ, e̯a, e̯o, i̯a, i̯e, i̯ə, i̯e, i̯o, øə, əe, əi, ə̯i	-	-	-	-	+	-	-	+	-	-	+	+	-	+	-	+	-	+	-	-	-	+	-
eːː	-	-	-	-	+	-	-	+	-	-	+	+	-	+	-	+	-	+	-	+	-	+	-
ẽː	-	-	-	-	+	-	-	+	-	-	+	+	+	+	-	+	-	+	-	-	-	+	-
æː	-	-	-	-	+	-	-	+	-	+	+	+	-	+	-	+	-	+	-	-	-	+	-
e̤ː, i̤e̤	-	-	-	-	+	-	-	+	+	-	+	+	-	+	-	+	-	+	-	-	-	+	-
b͡ç, c, cç, kʲ, qʲ, ɟ̊	-	-	-	+	-	-	-	-	-	-	-	-	-	-	-	-	-	-	-	-	+	-	-
b͡ʝ, ɟ, ɟʝ, ɡʲ	-	-	-	+	-	-	-	-	-	-	-	-	-	-	-	-	-	-	-	-	+	+	-
ʄ̊	-	-	-	+	-	-	-	-	-	-	-	-	-	-	-	-	+	-	-	-	+	-	-
ɠʲ, ʄ	-	-	-	+	-	-	-	-	-	-	-	-	-	-	-	-	+	-	-	-	+	+	-
c	-	-	-	+	-	-	-	-	-	-	-	-	-	-	-	+	-	-	-	-	+	-	-
ɟ	-	-	-	+	-	-	-	-	-	-	-	-	-	-	-	+	-	-	-	-	+	+	-
cç’, c’, k’ʲ, q’ʲ	-	-	-	+	-	-	-	-	-	-	-	-	-	-	-	+	+	-	-	-	+	-	-
c, ᵑc	-	-	-	+	-	-	-	-	-	-	-	-	+	-	-	-	-	-	-	-	+	-	-
ɟ	-	-	-	+	-	-	-	-	-	-	-	-	+	-	-	-	-	-	-	-	+	+	-
ɲ̊	-	-	-	+	-	-	-	-	-	-	-	-	+	+	-	-	-	-	-	-	+	-	-
ɲ, ᵐɲ	-	-	-	+	-	-	-	-	-	-	-	-	+	+	-	-	-	-	-	-	+	+	-
xʲ, ç, χʲ	-	-	-	+	-	-	-	-	-	-	-	+	-	-	-	-	-	-	-	-	+	-	-
ɣʲ, ʁʲ, ʝ	-	-	-	+	-	-	-	-	-	-	-	+	-	-	-	-	-	-	-	-	+	+	-
ʝ’	-	-	-	+	-	-	-	-	-	-	-	+	-	-	-	+	+	-	-	-	+	+	-
j, ĵ	-	-	-	+	-	-	-	-	-	-	-	+	-	+	-	-	-	-	-	-	-	+	-
ʎ̊	-	-	-	+	-	-	-	-	-	-	-	+	-	+	-	-	-	-	-	-	+	-	-
ʎ	-	-	-	+	-	-	-	-	-	-	-	+	-	+	-	-	-	+	-	-	+	+	-
j, jʰ	-	-	-	+	-	-	-	-	-	-	-	+	-	+	-	+	-	-	-	-	-	+	-
j’, ʔj	-	-	-	+	-	-	-	-	-	-	-	+	-	+	-	+	+	-	-	-	-	+	-
j̃	-	-	-	+	-	-	-	-	-	-	-	+	+	+	-	-	-	-	-	-	-	+	-
i̯, ɪ	-	-	-	+	-	-	-	-	-	-	+	+	-	+	-	-	-	+	-	-	-	+	-
ɪ̃	-	-	-	+	-	-	-	-	-	-	+	+	+	+	-	-	-	+	-	-	-	+	-
ɦʲ	-	-	-	+	-	-	-	-	-	+	-	+	-	+	-	-	-	-	-	-	-	+	-
cçʰ, cʰ, kʰʲ	-	-	-	+	-	-	-	-	+	-	-	-	-	-	-	+	-	-	-	-	+	-	-
ɟʰ	-	-	-	+	-	-	-	-	+	-	-	-	-	-	-	+	-	-	-	-	+	+	-
ʈʲʰ	-	-	-	+	-	-	-	-	+	-	-	-	-	-	+	+	-	-	-	-	+	-	-
ɲ̊ʰ	-	-	-	+	-	-	-	-	+	-	-	-	+	+	-	+	-	-	-	-	+	-	-
ɲʰ	-	-	-	+	-	-	-	-	+	-	-	-	+	+	-	+	-	-	-	-	+	+	-

	anterior	labial	back	high	ATR	lateral	distributed	LONG	spread	low	syllabic	continuant	nasal	sonorant	coronal	tense	constr	vocalic	strident	EXTRA	consonantal	voice	round
j̥	-	-	-	+	-	-	-	-	+	-	-	+	-	+	-	-	-	-	-	-	-	-	-
ɪ̥	-	-	-	+	-	-	-	-	+	-	+	+	-	+	-	-	-	-	-	-	-	-	-
ḧʲ	-	-	-	+	-	-	-	-	+	+	-	+	-	+	-	-	-	-	-	-	-	-	-
ɲː	-	-	-	+	-	-	-	+	-	-	-	-	+	+	-	-	-	-	-	-	+	+	-
jː	-	-	-	+	-	-	-	+	-	-	-	+	-	+	-	-	-	-	-	-	-	+	-
ɪː, ʊʏ̯	-	-	-	+	-	-	-	+	-	-	+	+	-	+	-	-	-	+	-	-	-	+	-
ĩː	-	-	-	+	-	-	-	+	-	-	+	+	+	+	-	-	-	+	-	-	-	+	-
cɕ	-	-	-	+	-	-	+	-	-	-	-	-	-	-	-	-	-	-	-	-	+	-	-
ɟʑ	-	-	-	+	-	-	+	-	-	-	-	-	-	-	-	-	-	-	-	-	+	+	-
cʃ, ɲ̥cʃ	-	-	-	+	-	-	+	-	-	-	-	-	-	-	-	-	-	-	+	-	+	-	-
c̀ɕ	-	-	-	+	-	-	+	-	-	-	-	-	-	-	-	+	-	-	-	-	+	-	-
ʃtʃ	-	-	-	+	-	-	+	-	-	-	-	-	-	-	+	-	-	-	-	-	+	-	-
tɕ, tʃ, tʃʱ, tʃʲ, t̪ʃ	-	-	-	+	-	-	+	-	-	-	-	-	-	-	+	-	-	-	+	-	+	-	-
dʑ, d̈ʒ, dʒʲ, d͡ʒg	-	-	-	+	-	-	+	-	-	-	-	-	-	-	+	-	-	-	+	-	+	+	-
tʃ, tʃ̀	-	-	-	+	-	-	+	-	-	-	-	-	-	-	+	+	-	-	+	-	+	-	-
dʒ	-	-	-	+	-	-	+	-	-	-	-	-	-	-	+	+	-	-	+	-	+	+	-
tʃʼ	-	-	-	+	-	-	+	-	-	-	-	-	-	-	+	+	+	-	+	-	+	-	-
d͡ʒ	-	-	-	+	-	-	+	-	-	-	-	-	-	-	+	+	+	-	+	-	+	+	-
t͡ʃk	-	-	-	+	-	-	+	-	-	-	-	-	-	+	+	-	-	-	+	-	+	-	-
tʃ, ᵐtʃ	-	-	-	+	-	-	+	-	-	-	-	-	+	-	+	-	-	-	+	-	+	-	-
dʒ, ᵐdʒ	-	-	-	+	-	-	+	-	-	-	-	-	+	-	+	-	-	-	+	-	+	+	-
t͡ʃʼ	-	-	-	+	-	-	+	-	-	-	-	-	+	-	+	+	+	-	+	-	+	-	-
ɲ͡ŋ	-	-	-	+	-	-	+	-	-	-	-	-	+	+	-	-	-	-	-	-	+	+	-
ɕ, ɲ̥ʃ, ʃ, ʃʲ	-	-	-	+	-	-	+	-	-	-	-	+	-	-	+	-	-	-	+	-	+	-	-
ʑ, ʒ̆, ʒʲ, ʒ̥	-	-	-	+	-	-	+	-	-	-	-	+	-	-	+	-	-	-	+	-	+	+	-
ʃ, ʃ̀	-	-	-	+	-	-	+	-	-	-	-	+	-	-	+	+	-	-	+	-	+	-	-
ʒ̤	-	-	-	+	-	-	+	-	-	-	-	+	-	-	+	+	-	-	+	-	+	+	-
ʃ̤ʼ	-	-	-	+	-	-	+	-	-	-	-	+	-	-	+	+	+	-	+	-	+	-	-
ʃ, ᵐʃ	-	-	-	+	-	-	+	-	-	-	-	+	+	-	+	-	-	-	+	-	+	-	-
ʒ	-	-	-	+	-	-	+	-	-	-	-	+	+	-	+	-	-	-	+	-	+	+	-
cɕʰ	-	-	-	+	-	-	+	-	+	-	-	-	-	-	-	+	-	-	-	-	+	-	-
tɕʰ, tʃʰ	-	-	-	+	-	-	+	-	+	-	-	-	-	-	+	+	-	-	+	-	+	-	-
dʒʰ	-	-	-	+	-	-	+	-	+	-	-	-	-	-	+	+	-	-	+	-	+	+	-
ɕʰ	-	-	-	+	-	-	+	-	+	-	-	+	-	-	+	+	-	-	+	-	+	-	-
ʒʰ	-	-	-	+	-	-	+	-	+	-	-	+	-	-	+	+	-	-	+	-	+	+	-
tːʃ	-	-	-	+	-	-	+	+	-	-	-	-	-	-	+	-	-	-	+	-	+	-	-
dːʒ	-	-	-	+	-	-	+	+	-	-	-	-	-	-	+	-	-	-	+	-	+	+	-
tːʃː	-	-	-	+	-	-	+	+	-	-	-	-	-	-	+	-	-	-	+	+	+	-	-
ʃː	-	-	-	+	-	-	+	+	-	-	-	+	-	-	+	-	-	-	+	-	+	-	-
ʒː	-	-	-	+	-	-	+	+	-	-	-	+	-	-	+	-	-	-	+	-	+	+	-
i, i̞, i̟, i̻, ɪ̞	-	-	-	+	+	-	-	-	-	-	+	+	-	+	-	+	-	+	-	-	-	+	-
iʔ, iʔi, ḭ	-	-	-	+	+	-	-	-	-	-	+	+	-	+	-	+	+	+	-	-	-	+	-
ĩ	-	-	-	+	+	-	-	-	-	-	+	+	+	+	-	+	-	+	-	-	-	+	-
ĩʔ, ĩʔĩ	-	-	-	+	+	-	-	-	-	-	+	+	+	+	-	+	+	+	-	-	-	+	-
i̥	-	-	-	+	+	-	-	-	+	-	+	+	-	+	-	+	-	-	-	-	-	-	-
i̤	-	-	-	+	+	-	-	-	+	-	+	+	-	+	-	+	-	+	-	-	-	+	-
i̤j, iː, i̞ː, je, jeː, ui, yi	-	-	-	+	+	-	-	+	-	-	+	+	-	+	-	+	-	+	-	-	-	+	-
iːː	-	-	-	+	+	-	-	+	-	-	+	+	-	+	-	+	-	+	-	+	-	+	-
ḭː	-	-	-	+	+	-	-	+	-	-	+	+	-	+	-	+	+	+	-	-	-	+	-
ĩː, jẽ	-	-	-	+	+	-	-	+	-	-	+	+	+	+	-	+	-	+	-	-	-	+	-
i̤ː	-	-	-	+	+	-	-	+	+	-	+	+	-	+	-	+	-	+	-	-	-	+	-

	anterior	labial	back	high	ATR	lateral	distributed	LONG	spread	low	syllabic	continuant	nasal	sonorant	coronal	tense	constr	vocalic	strident	EXTRA	consonantal	voice	round
q, qχ	-	-	+	-	-	-	-	-	-	-	-	-	-	-	-	-	-	-	-	-	+	-	-
ɢ	-	-	+	-	-	-	-	-	-	-	-	-	-	-	-	-	-	-	-	-	+	+	-
ʛ	-	-	+	-	-	-	-	-	-	-	-	-	-	-	-	-	+	-	-	-	+	+	-
q’	-	-	+	-	-	-	-	-	-	-	-	-	-	-	-	+	+	-	-	-	+	-	-
ɴ	-	-	+	-	-	-	-	-	-	-	-	-	+	+	-	-	-	-	-	-	+	+	-
ɴ’	-	-	+	-	-	-	-	-	-	-	-	-	+	+	-	+	+	-	-	-	+	+	-
χ	-	-	+	-	-	-	-	-	-	-	-	+	-	-	-	-	-	-	-	-	+	-	-
ʁ	-	-	+	-	-	-	-	-	-	-	-	+	-	-	-	-	-	-	-	-	+	+	-
χ̀	-	-	+	-	-	-	-	-	-	-	-	+	-	-	-	+	-	-	-	-	+	-	-
qɰ	-	-	+	-	-	-	-	-	-	-	-	+	-	+	-	-	-	-	-	-	-	+	-
ʀ	-	-	+	-	-	-	-	-	-	-	-	+	-	+	-	-	-	+	-	-	+	+	-
ʀ’	-	-	+	-	-	-	-	-	-	-	-	+	-	+	-	+	+	+	-	-	+	+	-
ə, ə̟, ɜ, ʌ, ʌ̟	-	-	+	-	-	-	-	-	-	-	+	+	-	+	-	-	-	+	-	-	-	+	-
ə̆, ʌ̆	-	-	+	-	-	-	-	-	-	-	+	+	-	+	-	-	-	+	-	+	-	+	-
ɚ	-	-	+	-	-	-	-	-	-	-	+	+	-	+	+	-	-	+	-	-	-	+	-
ə̃, ʌ̃	-	-	+	-	-	-	-	-	-	-	+	+	+	+	-	-	-	+	-	-	-	+	-
qˁ	-	-	+	-	-	-	-	-	-	+	-	-	-	-	-	-	-	-	-	-	+	-	-
ɢˁ	-	-	+	-	-	-	-	-	-	+	-	-	-	-	-	-	-	-	-	-	+	+	-
q’ˁ, qˁ’	-	-	+	-	-	-	-	-	-	+	-	-	-	-	-	+	+	-	-	-	+	-	-
ħ, χˁ	-	-	+	-	-	-	-	-	-	+	-	+	-	-	-	-	-	-	-	-	+	-	-
ʁˁ, ʕ	-	-	+	-	-	-	-	-	-	+	-	+	-	-	-	-	-	-	-	-	+	+	-
ʕ’	-	-	+	-	-	-	-	-	-	+	-	+	-	-	-	+	+	-	-	-	+	+	-
a+, a, aˁ, a̟, ɑ, əˁ	-	-	+	-	-	-	-	-	-	+	+	+	-	+	-	-	-	+	-	-	-	+	-
ă	-	-	+	-	-	-	-	-	-	+	+	+	-	+	-	-	-	+	-	+	-	+	-
aʔ, aʔa, aˁʔ, a̰	-	-	+	-	-	-	-	-	-	+	+	+	-	+	-	-	+	+	-	-	-	+	-
ã, ãˁ, ɑ̃	-	-	+	-	-	-	-	-	-	+	+	+	+	+	-	-	-	+	-	-	-	+	-
ãʔ, ãʔã	-	-	+	-	-	-	-	-	-	+	+	+	+	+	-	-	+	+	-	-	-	+	-
qʰ	-	-	+	-	-	-	-	-	+	-	-	-	-	-	-	+	-	-	-	-	+	-	-
ʌ̤	-	-	+	-	-	-	-	-	+	-	+	+	-	+	-	-	-	+	-	-	-	+	-
ʔˁ	-	-	+	-	-	-	-	-	+	+	-	-	-	+	-	-	+	-	-	-	-	-	-
hˁ	-	-	+	-	-	-	-	-	+	+	-	+	-	+	-	-	-	-	-	-	-	-	-
ḁ	-	-	+	-	-	-	-	-	+	+	+	+	-	+	-	-	-	-	-	-	-	-	-
a̤, a̤ˁ	-	-	+	-	-	-	-	-	+	+	+	+	-	+	-	-	-	+	-	-	-	+	-
a̤ʔ	-	-	+	-	-	-	-	-	+	+	+	+	-	+	-	-	+	+	-	-	-	+	-
ã̤, ã̤ˁ	-	-	+	-	-	-	-	-	+	+	+	+	+	+	-	-	-	+	-	-	-	+	-
ã̤ʔ	-	-	+	-	-	-	-	-	+	+	+	+	+	+	-	-	+	+	-	-	-	+	-
qː	-	-	+	-	-	-	-	+	-	-	-	-	-	-	-	-	-	-	-	-	+	-	-
χː	-	-	+	-	-	-	-	+	-	-	-	+	-	-	-	-	-	-	-	-	+	-	-
ʁː	-	-	+	-	-	-	-	+	-	-	-	+	-	-	-	-	-	-	-	-	+	+	-
aʊ, ɔj, əː, ɜː, ʌː	-	-	+	-	-	-	-	+	-	-	+	+	-	+	-	-	-	+	-	-	-	+	-
ə̃ː, ʌ̃ː	-	-	+	-	-	-	-	+	-	-	+	+	+	+	-	-	-	+	-	-	-	+	-
aː, ɑː	-	-	+	-	-	-	-	+	-	+	+	+	-	+	-	-	-	+	-	-	-	+	-
aːː	-	-	+	-	-	-	-	+	-	+	+	+	-	+	-	-	-	+	-	+	-	+	-
a̰ː	-	-	+	-	-	-	-	+	-	+	+	+	-	+	-	-	+	+	-	-	-	+	-
ãː	-	-	+	-	-	-	-	+	-	+	+	+	+	+	-	-	-	+	-	-	-	+	-
ʌ̤ː	-	-	+	-	-	-	-	+	+	-	+	+	-	+	-	-	-	+	-	-	-	+	-
ə̟, ɤ	-	-	+	-	+	-	-	-	-	-	+	+	-	+	-	+	-	+	-	-	-	+	-
a̟, a̠, eˁ, ɐ	-	-	+	-	+	-	-	-	-	+	+	+	-	+	-	+	-	+	-	-	-	+	-
aj, au, oə, o̹a, uə, əo, əu, ɤː, ɯə	-	-	+	-	+	-	-	+	-	-	+	+	-	+	-	+	-	+	-	-	-	+	-
a̟ː, a̠ː, ɐː	-	-	+	-	+	-	-	+	-	+	+	+	-	+	-	+	-	+	-	-	-	+	-

	anterior	labial	back	high	ATR	lateral	distributed	LONG	spread	low	syllabic	continuant	nasal	sonorant	coronal	tense	constr	vocalic	strident	EXTRA	consonantal	voice	round
k, k̂, ɡ̊	-	-	+	+	-	-	-	-	-	-	-	-	-	-	-	-	-	-	-	-	+	-	-
ɡ, ɡ̂	-	-	+	+	-	-	-	-	-	-	-	-	-	-	-	-	-	-	-	-	+	+	-
ʛ̥	-	-	+	+	-	-	-	-	-	-	-	-	-	-	-	-	+	-	-	-	+	-	-
ʛ	-	-	+	+	-	-	-	-	-	-	-	-	-	-	-	-	+	-	-	-	+	+	-
k, k̀	-	-	+	+	-	-	-	-	-	-	-	-	-	-	-	+	-	-	-	-	+	-	-
ɡ	-	-	+	+	-	-	-	-	-	-	-	-	-	-	-	+	-	-	-	-	+	+	-
kx’, k’, ɡkx’, ʔk	-	-	+	+	-	-	-	-	-	-	-	-	-	-	-	+	+	-	-	-	+	-	-
kᵑ, ᵑk, ᵑɡ̊	-	-	+	+	-	-	-	-	-	-	-	-	+	-	-	-	-	-	-	-	+	-	-
ᵑɡ	-	-	+	+	-	-	-	-	-	-	-	-	+	-	-	-	-	-	-	-	+	+	-
ᵑkx’	-	-	+	+	-	-	-	-	-	-	-	-	+	-	-	+	+	-	-	-	+	-	-
ŋ̊	-	-	+	+	-	-	-	-	-	-	-	-	+	+	-	-	-	-	-	-	+	-	-
ŋ	-	-	+	+	-	-	-	-	-	-	-	-	+	+	-	-	-	-	-	-	+	+	-
ŋ’	-	-	+	+	-	-	-	-	-	-	-	-	+	+	-	+	+	-	-	-	+	+	-
x	-	-	+	+	-	-	-	-	-	-	-	+	-	-	-	-	-	-	-	-	+	-	-
ɣ	-	-	+	+	-	-	-	-	-	-	-	+	-	-	-	-	-	-	-	-	+	+	-
x, x̀	-	-	+	+	-	-	-	-	-	-	-	+	-	-	-	+	-	-	-	-	+	-	-
ɣ, ɣ̀	-	-	+	+	-	-	-	-	-	-	-	+	-	-	-	+	-	-	-	-	+	+	-
x’	-	-	+	+	-	-	-	-	-	-	-	+	-	-	-	+	+	-	-	-	+	-	-
ɣ’	-	-	+	+	-	-	-	-	-	-	-	+	-	-	-	+	+	-	-	-	+	+	-
ɰ	-	-	+	+	-	-	-	-	-	-	-	+	-	+	-	-	-	-	-	-	-	+	-
ɰ’	-	-	+	+	-	-	-	-	-	-	-	+	-	+	-	+	+	-	-	-	-	+	-
ɨ̞	-	-	+	+	-	-	-	-	-	-	+	+	-	+	-	-	-	+	-	-	-	+	-
cˤ, kˤ	-	-	+	+	-	-	-	-	-	+	-	-	-	-	-	-	-	-	-	-	+	-	-
ɡˤ	-	-	+	+	-	-	-	-	-	+	-	-	-	-	-	-	-	-	-	-	+	+	-
kxʰ, kʰ	-	-	+	+	-	-	-	-	+	-	-	-	-	-	-	+	-	-	-	-	+	-	-
ɡʰ	-	-	+	+	-	-	-	-	+	-	-	-	-	-	-	+	-	-	-	-	+	+	-
ŋ̊ʰ	-	-	+	+	-	-	-	-	+	-	-	-	+	+	-	+	-	-	-	-	+	-	-
ŋʰ	-	-	+	+	-	-	-	-	+	-	-	-	+	+	-	+	-	-	-	-	+	+	-
xʰ	-	-	+	+	-	-	-	-	+	-	-	+	-	-	-	+	-	-	-	-	+	-	-
ɣʰ	-	-	+	+	-	-	-	-	+	-	-	+	-	-	-	+	-	-	-	-	+	+	-
kː	-	-	+	+	-	-	-	+	-	-	-	-	-	-	-	-	-	-	-	-	+	-	-
ɡː	-	-	+	+	-	-	-	+	-	-	-	-	-	-	-	-	-	-	-	-	+	+	-
ŋː	-	-	+	+	-	-	-	+	-	-	-	-	+	+	-	-	-	-	-	-	+	+	-
xː	-	-	+	+	-	-	-	+	-	-	-	+	-	-	-	-	-	-	-	-	+	-	-
ǂ, ǂq, ǂx	-	-	+	+	-	-	+	-	-	-	-	-	-	-	+	-	-	-	-	-	+	-	-
ɡǂ, ɡǂkx, ɡǂx	-	-	+	+	-	-	+	-	-	-	-	-	-	-	+	-	-	-	-	-	+	+	-
ǂkxʔ, ǂqʔ, ǂʔ	-	-	+	+	-	-	+	-	-	-	-	-	-	-	+	+	+	-	-	-	+	-	-
mǂG, nǂ, ǂn, ǂŋ̊	-	-	+	+	-	-	+	-	-	-	-	-	+	-	+	-	-	-	-	-	+	-	-
nɡǂ	-	-	+	+	-	-	+	-	-	-	-	-	+	-	+	-	-	-	-	-	+	+	-
ʔǂn	-	-	+	+	-	-	+	-	-	-	-	-	+	-	+	+	+	-	-	-	+	-	-
z̻ˤ	-	-	+	+	-	-	+	-	-	+	-	+	-	-	+	-	-	-	+	-	+	+	-
ǂqʰ	-	-	+	+	-	-	+	-	+	-	-	-	-	-	+	-	-	-	-	-	+	-	-
ǂʰ	-	-	+	+	-	-	+	-	+	-	-	-	-	-	+	+	-	-	-	-	+	-	-
ɡǂʰ	-	-	+	+	-	-	+	-	+	-	-	-	-	-	+	+	-	-	-	-	+	+	-
kl’	-	-	+	+	-	+	-	-	-	-	-	-	-	-	-	+	+	-	-	-	+	-	-
ɨ, ɯ	-	-	+	+	+	-	-	-	-	-	+	+	-	+	-	+	-	+	-	-	-	+	-
ɨʔɨ	-	-	+	+	+	-	-	-	-	-	+	+	-	+	-	+	+	+	-	-	-	+	-
ɨ˞	-	-	+	+	+	-	-	-	-	-	+	+	-	+	+	+	-	+	-	-	-	+	-
ɨ̃, ɯ̃	-	-	+	+	+	-	-	-	-	-	+	+	+	+	-	+	-	+	-	-	-	+	-
ɨ̃ʔɨ̃	-	-	+	+	+	-	-	-	-	-	+	+	+	+	-	+	+	+	-	-	-	+	-
iˤ	-	-	+	+	+	-	-	-	-	+	+	+	-	+	-	+	-	+	-	-	-	+	-

	anterior	labial	back	high	ATR	lateral	distributed	LONG	spread	low	syllabic	continuant	nasal	sonorant	coronal	tense	constr	vocalic	strident	EXTRA	consonantal	voice	round
ɨː, ɯː	-	-	+	+	+	-	-	+	-	-	+	+	-	+	-	+	-	+	-	-	-	+	-
ɨːː	-	-	+	+	+	-	-	+	-	-	+	+	-	+	-	+	-	+	-	+	-	+	-
ɨ̃ː	-	-	+	+	+	-	-	+	-	-	+	+	+	+	-	+	-	+	-	-	-	+	-
dɣw	-	+	-	-	-	-	-	-	-	-	-	-	-	-	-	-	-	-	-	-	+	+	+
ɳʷ	-	+	-	-	-	-	-	-	-	-	-	-	+	+	+	-	-	-	-	-	+	+	+
œ	-	+	-	-	-	-	-	-	-	-	+	+	-	+	-	-	-	+	-	-	-	+	+
œ̃	-	+	-	-	-	-	-	-	-	-	+	+	+	+	-	-	-	+	-	-	-	+	+
ɦʷ	-	+	-	-	-	-	-	-	-	+	-	+	-	+	-	-	-	-	-	-	-	+	+
ʔʷ	-	+	-	-	-	-	-	-	+	+	-	-	-	+	-	+	+	-	-	-	-	-	+
hʷ	-	+	-	-	-	-	-	-	+	+	-	+	-	+	-	-	-	-	-	-	-	-	+
œu, œː	-	+	-	-	-	-	-	+	-	-	+	+	-	+	-	-	-	+	-	-	-	+	+
hʷː	-	+	-	-	-	-	-	+	+	+	-	+	-	+	-	-	-	-	-	-	-	-	+
ø	-	+	-	-	+	-	-	-	-	-	+	+	-	+	-	+	-	+	-	-	-	+	+
ø̆	-	+	-	-	+	-	-	-	-	-	+	+	-	+	-	+	-	+	-	+	-	+	+
oi, øː	-	+	-	-	+	-	-	+	-	-	+	+	-	+	-	+	-	+	-	-	-	+	+
cʷ	-	+	-	+	-	-	-	-	-	-	-	-	-	-	-	-	-	-	-	-	+	-	+
ɟʷ	-	+	-	+	-	-	-	-	-	-	-	-	-	-	-	-	-	-	-	-	+	+	+
ɲɥ	-	+	-	+	-	-	-	-	-	-	-	-	+	+	-	-	-	-	-	-	+	+	+
çʷ	-	+	-	+	-	-	-	-	-	-	-	+	-	-	-	-	-	-	-	-	+	-	+
jʷ, ɥ	-	+	-	+	-	-	-	-	-	-	-	+	-	+	-	-	-	-	-	-	-	+	+
ʏ	-	+	-	+	-	-	-	-	-	-	+	+	-	+	-	-	-	+	-	-	-	+	+
ɥ̥	-	+	-	+	-	-	-	-	+	-	-	+	-	+	-	-	-	-	-	-	-	-	+
tɕɥ, ʃ͡kʷ	-	+	-	+	-	-	+	-	-	-	-	-	-	-	+	-	-	-	-	-	+	-	+
d͡ʒɣʷ	-	+	-	+	-	-	+	-	-	-	-	-	-	-	+	-	-	-	-	-	+	+	+
tʃʷ, tʃ͡kʷ	-	+	-	+	-	-	+	-	-	-	-	-	-	-	+	-	-	-	+	-	+	-	+
dʑɥ, dʒʷ	-	+	-	+	-	-	+	-	-	-	-	-	-	-	+	-	-	-	+	-	+	+	+
tʃʷ’	-	+	-	+	-	-	+	-	-	-	-	-	-	-	+	+	+	-	+	-	+	-	+
ɲ͡ŋw	-	+	-	+	-	-	+	-	-	-	-	-	+	+	-	-	-	-	-	-	+	+	+
ʒ͡ɣw	-	+	-	+	-	-	+	-	-	-	-	+	-	-	+	-	-	-	-	-	+	+	+
ɕɥ, ʃʷ, ʃ̥ʷ	-	+	-	+	-	-	+	-	-	-	-	+	-	-	+	-	-	-	+	-	+	-	+
ʒʷ, ʒ̥ʷ	-	+	-	+	-	-	+	-	-	-	-	+	-	-	+	-	-	-	+	-	+	+	+
tʃʰʷ	-	+	-	+	-	-	+	-	+	-	-	-	-	-	+	+	-	-	+	-	+	-	+
y	-	+	-	+	+	-	-	-	-	-	+	+	-	+	-	+	-	+	-	-	-	+	+
ỹ	-	+	-	+	+	-	-	-	-	-	+	+	+	+	-	+	-	+	-	-	-	+	+
uw, yː	-	+	-	+	+	-	-	+	-	-	+	+	-	+	-	+	-	+	-	-	-	+	+
qʷ	-	+	+	-	-	-	-	-	-	-	-	-	-	-	-	-	-	-	-	-	+	-	+
qʷ’, q’ʷ	-	+	+	-	-	-	-	-	-	-	-	-	-	-	-	+	+	-	-	-	+	-	+
χʷ	-	+	+	-	-	-	-	-	-	-	-	+	-	-	-	-	-	-	-	-	+	-	+
ʁʷ	-	+	+	-	-	-	-	-	-	-	-	+	-	-	-	-	-	-	-	-	+	+	+
χ̀ʷ	-	+	+	-	-	-	-	-	-	-	-	+	-	-	-	+	-	-	-	-	+	-	+
o̞, ɔ, ɔ̞	-	+	+	-	-	-	-	-	-	-	+	+	-	+	-	-	-	+	-	-	-	+	+
ɔʔ	-	+	+	-	-	-	-	-	-	-	+	+	-	+	-	-	+	+	-	-	-	+	+
ɔ̃	-	+	+	-	-	-	-	-	-	-	+	+	+	+	-	-	-	+	-	-	-	+	+
ɔ̃ʔ	-	+	+	-	-	-	-	-	-	-	+	+	+	+	-	-	+	+	-	-	-	+	+
qˤʷ	-	+	+	-	-	-	-	-	-	+	-	-	-	-	-	+	-	-	-	-	+	-	+
q’ˤʷ	-	+	+	-	-	-	-	-	-	+	-	-	-	-	-	+	+	-	-	-	+	-	+
ħʷ, χˤʷ	-	+	+	-	-	-	-	-	-	+	-	+	-	-	-	-	-	-	-	-	+	-	+
ʁˤʷ, ʕʷ	-	+	+	-	-	-	-	-	-	+	-	+	-	-	-	-	-	-	-	-	+	+	+
ʕ’ʷ	-	+	+	-	-	-	-	-	-	+	-	+	-	-	-	+	+	-	-	-	+	+	+
ɒ	-	+	+	-	-	-	-	-	-	+	+	+	-	+	-	-	-	+	-	-	-	+	+
qʰʷ	-	+	+	-	-	-	-	-	+	-	-	-	-	-	-	+	-	-	-	-	+	-	+
ɔ̥	-	+	+	-	-	-	-	-	+	-	+	+	-	+	-	-	-	-	-	-	-	-	+
qʷː	-	+	+	-	-	-	-	+	-	-	-	-	-	-	-	-	-	-	-	-	+	-	+
uɔ, ɔː, ɔ̞ː, ʊɔ	-	+	+	-	-	-	-	+	-	-	+	+	-	+	-	-	-	+	-	-	-	+	+

	anterior	labial	back	high	ATR	lateral	distributed	LONG	spread	low	syllabic	continuant	nasal	sonorant	coronal	tense	constr	vocalic	strident	EXTRA	consonantal	voice	round
ɔ̃ː	-	+	+	-	-	-	-	+	-	-	+	+	+	+	-	-	-	+	-	-	-	+	+
ɒː	-	+	+	-	-	-	-	+	-	+	+	+	-	+	-	-	-	+	-	-	-	+	+
o, omf, o̝, ɔ̝, ɵ	-	+	+	-	+	-	-	-	-	-	+	+	-	+	-	+	-	+	-	-	-	+	+
ŏ	-	+	+	-	+	-	-	-	-	-	+	+	-	+	-	+	-	+	-	+	-	+	+
oʔ, oʔo	-	+	+	-	+	-	-	-	-	-	+	+	-	+	-	+	+	+	-	-	-	+	+
õ, õ̝	-	+	+	-	+	-	-	-	-	-	+	+	+	+	-	+	-	+	-	-	-	+	+
õʔ	-	+	+	-	+	-	-	-	-	-	+	+	+	+	-	+	+	+	-	-	-	+	+
oˤ	-	+	+	-	+	-	-	-	-	+	+	+	-	+	-	+	-	+	-	-	-	+	+
õˤ	-	+	+	-	+	-	-	-	-	+	+	+	+	+	-	+	-	+	-	-	-	+	+
o̥	-	+	+	-	+	-	-	-	+	-	+	+	-	+	-	+	-	-	-	-	-	-	+
o̤	-	+	+	-	+	-	-	-	+	-	+	+	-	+	-	+	-	+	-	-	-	+	+
o̤ʔ	-	+	+	-	+	-	-	-	+	-	+	+	-	+	-	+	+	+	-	-	-	+	+
õ̤	-	+	+	-	+	-	-	-	+	-	+	+	+	+	-	+	-	+	-	-	-	+	+
õ̤ʔ	-	+	+	-	+	-	-	-	+	-	+	+	+	+	-	+	+	+	-	-	-	+	+
o̤ˤ	-	+	+	-	+	-	-	-	+	+	+	+	-	+	-	+	-	+	-	-	-	+	+
ou, oʊ, oː, o̝ː, o̯u, uo, u̯o	-	+	+	-	+	-	-	+	-	-	+	+	-	+	-	+	-	+	-	-	-	+	+
oːː	-	+	+	-	+	-	-	+	-	-	+	+	-	+	-	+	-	+	-	+	-	+	+
õː	-	+	+	-	+	-	-	+	-	-	+	+	+	+	-	+	-	+	-	-	-	+	+
o̤ː, ṳo̤	-	+	+	-	+	-	-	+	+	-	+	+	-	+	-	+	-	+	-	-	-	+	+
kʷ, kʷʱ	-	+	+	+	-	-	-	-	-	-	-	-	-	-	-	-	-	-	-	-	+	-	+
gʷ	-	+	+	+	-	-	-	-	-	-	-	-	-	-	-	-	-	-	-	-	+	+	+
ɠʷ	-	+	+	+	-	-	-	-	-	-	-	-	-	-	-	-	+	-	-	-	+	+	+
k̀ʷ	-	+	+	+	-	-	-	-	-	-	-	-	-	-	-	+	-	-	-	-	+	-	+
kʷ’, k’ʷ	-	+	+	+	-	-	-	-	-	-	-	-	-	-	-	+	+	-	-	-	+	-	+
ᵑgʷ	-	+	+	+	-	-	-	-	-	-	-	-	+	-	-	-	-	-	-	-	+	+	+
ŋʷ	-	+	+	+	-	-	-	-	-	-	-	-	+	+	-	-	-	-	-	-	+	+	+
xʷ	-	+	+	+	-	-	-	-	-	-	-	+	-	-	-	-	-	-	-	-	+	-	+
ɣʷ	-	+	+	+	-	-	-	-	-	-	-	+	-	-	-	-	-	-	-	-	+	+	+
x̀ʷ	-	+	+	+	-	-	-	-	-	-	-	+	-	-	-	+	-	-	-	-	+	-	+
u̞, ʊ	-	+	+	+	-	-	-	-	-	-	+	+	-	+	-	-	-	+	-	-	-	+	+
ʊ̃	-	+	+	+	-	-	-	-	-	-	+	+	+	+	-	-	-	+	-	-	-	+	+
kʰʷ	-	+	+	+	-	-	-	-	+	-	-	-	-	-	-	+	-	-	-	-	+	-	+
ʊ̥	-	+	+	+	-	-	-	-	+	-	+	+	-	+	-	-	-	-	-	-	-	-	+
ṳ̃	-	+	+	+	-	-	-	-	+	-	+	+	+	+	-	-	-	+	-	-	-	+	+
kʷː	-	+	+	+	-	-	-	+	-	-	-	-	-	-	-	-	-	-	-	-	+	-	+
gʷː	-	+	+	+	-	-	-	+	-	-	-	-	-	-	-	-	-	-	-	-	+	+	+
ʊː	-	+	+	+	-	-	-	+	-	-	+	+	-	+	-	-	-	+	-	-	-	+	+
ʊ̃ː	-	+	+	+	-	-	-	+	-	-	+	+	+	+	-	-	-	+	-	-	-	+	+
u, u̞, ʉ, ʊ̝	-	+	+	+	+	-	-	-	-	-	+	+	-	+	-	+	-	+	-	-	-	+	+
uʔ, uʔu, ṵ	-	+	+	+	+	-	-	-	-	-	+	+	-	+	-	+	+	+	-	-	-	+	+
ũ	-	+	+	+	+	-	-	-	-	-	+	+	+	+	-	+	-	+	-	-	-	+	+
ũʔ, ũʔũ	-	+	+	+	+	-	-	-	-	-	+	+	+	+	-	+	+	+	-	-	-	+	+
uˤ	-	+	+	+	+	-	-	-	-	+	+	+	-	+	-	+	-	+	-	-	-	+	+
uˤʔ	-	+	+	+	+	-	-	-	-	+	+	+	-	+	-	+	+	+	-	-	-	+	+
ũˤ	-	+	+	+	+	-	-	-	-	+	+	+	+	+	-	+	-	+	-	-	-	+	+
u̥	-	+	+	+	+	-	-	-	+	-	+	+	-	+	-	+	-	-	-	-	-	-	+
ṳ	-	+	+	+	+	-	-	-	+	-	+	+	-	+	-	+	-	+	-	-	-	+	+
ṳʔ	-	+	+	+	+	-	-	-	+	-	+	+	-	+	-	+	+	+	-	-	-	+	+
ṳˤ	-	+	+	+	+	-	-	-	+	+	+	+	-	+	-	+	-	+	-	-	-	+	+
uː, u̞ː, ʉ̞ː	-	+	+	+	+	-	-	+	-	-	+	+	-	+	-	+	-	+	-	-	-	+	+
uːː	-	+	+	+	+	-	-	+	-	-	+	+	-	+	-	+	-	+	-	+	-	+	+
ṵː	-	+	+	+	+	-	-	+	-	-	+	+	-	+	-	+	+	+	-	-	-	+	+

	anterior	labial	back	high	ATR	lateral	distributed	LONG	spread	low	syllabic	continuant	nasal	sonorant	coronal	tense	constr	vocalic	strident	EXTRA	consonantal	voice	round
ũː	-	+	+	+	+	-	-	+	-	-	+	+	+	+	-	+	-	+	-	-	-	+	+
ṳː	-	+	+	+	+	-	-	+	+	-	+	+	-	+	-	+	-	+	-	-	-	+	+
dzx, p͡t, tsx	+	-	-	-	-	-	-	-	-	-	-	-	-	-	-	-	-	-	-	-	+	-	-
b͡d, dβ	+	-	-	-	-	-	-	-	-	-	-	-	-	-	-	-	-	-	-	-	+	+	-
dzɣw	+	-	-	-	-	-	-	-	-	-	-	-	-	-	-	-	-	-	-	-	+	+	+
ps, p͡ts	+	-	-	-	-	-	-	-	-	-	-	-	-	-	-	-	-	-	+	-	+	-	-
bz, b͡dz	+	-	-	-	-	-	-	-	-	-	-	-	-	-	-	-	-	-	+	-	+	+	-
r͡ɣw	+	-	-	-	-	-	-	-	-	-	-	-	-	-	-	-	-	-	+	-	+	+	+
dz’kx’, d’kx’, ts’kx’, t’kx’	+	-	-	-	-	-	-	-	-	-	-	-	-	-	-	+	+	-	-	-	+	-	-
b͡dɗ	+	-	-	-	-	-	-	-	-	-	-	-	-	-	-	+	+	-	+	-	+	+	-
d̥, t, tr, tʱ, t̂, t̪θ, tθ	+	-	-	-	-	-	-	-	-	-	-	-	-	-	+	-	-	-	-	-	+	-	-
d, dð, d̂, d̪ð	+	-	-	-	-	-	-	-	-	-	-	-	-	-	+	-	-	-	-	-	+	+	-
ts, tz	+	-	-	-	-	-	-	-	-	-	-	-	-	-	+	-	-	-	+	-	+	-	-
dz	+	-	-	-	-	-	-	-	-	-	-	-	-	-	+	-	-	-	+	-	+	+	-
ɗ	+	-	-	-	-	-	-	-	-	-	-	-	-	-	+	-	+	-	-	-	+	+	-
t, t̀	+	-	-	-	-	-	-	-	-	-	-	-	-	-	+	+	-	-	-	-	+	-	-
d, d̏	+	-	-	-	-	-	-	-	-	-	-	-	-	-	+	+	-	-	-	-	+	+	-
ts, ts̀	+	-	-	-	-	-	-	-	-	-	-	-	-	-	+	+	-	-	+	-	+	-	-
dz	+	-	-	-	-	-	-	-	-	-	-	-	-	-	+	+	-	-	+	-	+	+	-
t’, tθ’, ʔt	+	-	-	-	-	-	-	-	-	-	-	-	-	-	+	+	+	-	-	-	+	-	-
d’, ɗ, ʔd	+	-	-	-	-	-	-	-	-	-	-	-	-	-	+	+	+	-	-	-	+	+	-
ts’, tz’	+	-	-	-	-	-	-	-	-	-	-	-	-	-	+	+	+	-	+	-	+	-	-
dz’	+	-	-	-	-	-	-	-	-	-	-	-	-	-	+	+	+	-	+	-	+	+	-
ɾ, ɾð	+	-	-	-	-	-	-	-	-	-	-	-	-	+	+	-	-	+	-	-	+	+	-
ɾ	+	-	-	-	-	-	-	-	-	-	-	-	-	+	+	+	-	+	-	-	+	+	-
tɸ	+	-	-	-	-	-	-	-	-	-	-	-	+	-	-	-	-	-	-	-	+	-	-
dβ	+	-	-	-	-	-	-	-	-	-	-	-	+	-	-	-	-	-	-	-	+	+	-
nd̥, t, tⁿ, ᵐt	+	-	-	-	-	-	-	-	-	-	-	-	+	-	+	-	-	-	-	-	+	-	-
d, ᵐd	+	-	-	-	-	-	-	-	-	-	-	-	+	-	+	-	-	-	-	-	+	+	-
ts, ᵐts	+	-	-	-	-	-	-	-	-	-	-	-	+	-	+	-	-	-	+	-	+	-	-
dz, ᵐdz	+	-	-	-	-	-	-	-	-	-	-	-	+	-	+	-	-	-	+	-	+	+	-
ts’	+	-	-	-	-	-	-	-	-	-	-	-	+	-	+	+	+	-	+	-	+	-	-
nŋw	+	-	-	-	-	-	-	-	-	-	-	-	+	+	-	-	-	-	-	-	+	+	+
n̥	+	-	-	-	-	-	-	-	-	-	-	-	+	+	+	-	-	-	-	-	+	-	-
n, n̂, n̩, n̩	+	-	-	-	-	-	-	-	-	-	-	-	+	+	+	-	-	-	-	-	+	+	-
ɾ̃	+	-	-	-	-	-	-	-	-	-	-	-	+	+	+	-	-	+	-	-	+	+	-
n̏	+	-	-	-	-	-	-	-	-	-	-	-	+	+	+	+	-	-	-	-	+	+	-
n’, ʔn	+	-	-	-	-	-	-	-	-	-	-	-	+	+	+	+	+	-	-	-	+	+	-
zʷ͡ɣw, z͡ɣw	+	-	-	-	-	-	-	-	-	-	-	+	-	-	-	-	-	-	-	-	+	+	+
s̪v	+	-	-	-	-	-	-	-	-	-	-	+	-	-	-	-	-	-	+	-	+	-	-
z̪v	+	-	-	-	-	-	-	-	-	-	-	+	-	-	-	-	-	-	+	-	+	+	-
θ̄	+	-	-	-	-	-	-	-	-	-	-	+	-	-	+	-	-	-	-	-	+	-	-
ð	+	-	-	-	-	-	-	-	-	-	-	+	-	-	+	-	-	-	-	-	+	+	-
n̥s, s, š, s̩	+	-	-	-	-	-	-	-	-	-	-	+	-	-	+	-	-	-	+	-	+	-	-
z	+	-	-	-	-	-	-	-	-	-	-	+	-	-	+	-	-	-	+	-	+	+	-
θ	+	-	-	-	-	-	-	-	-	-	-	+	-	-	+	+	-	-	-	-	+	-	-
s, s̀	+	-	-	-	-	-	-	-	-	-	-	+	-	-	+	+	-	-	+	-	+	-	-
z	+	-	-	-	-	-	-	-	-	-	-	+	-	-	+	+	-	-	+	-	+	+	-

	anterior	labial	back	high	ATR	lateral	distributed	LONG	spread	low	syllabic	continuant	nasal	sonorant	coronal	tense	constr	vocalic	strident	EXTRA	consonantal	voice	round
θ’	+	-	-	-	-	-	-	-	-	-	-	+	-	-	+	+	+	-	-	-	+	-	-
s’	+	-	-	-	-	-	-	-	-	-	-	+	-	-	+	+	+	-	+	-	+	-	-
z’	+	-	-	-	-	-	-	-	-	-	-	+	-	-	+	+	+	-	+	-	+	+	-
r̥	+	-	-	-	-	-	-	-	-	-	-	+	-	+	+	-	-	-	-	-	+	-	-
r, r̟, ɹ	+	-	-	-	-	-	-	-	-	-	-	+	-	+	+	-	-	+	-	-	+	+	-
ř	+	-	-	-	-	-	-	-	-	-	-	+	-	+	+	-	-	+	+	-	+	+	-
r̥	+	-	-	-	-	-	-	-	-	-	-	+	-	+	+	+	-	-	-	-	+	-	-
r, ɹ	+	-	-	-	-	-	-	-	-	-	-	+	-	+	+	+	-	+	-	-	+	+	-
n̟s̟, s	+	-	-	-	-	-	-	-	-	-	-	+	+	-	+	-	-	-	+	-	+	-	-
z	+	-	-	-	-	-	-	-	-	-	-	+	+	-	+	-	-	-	+	-	+	+	-
r	+	-	-	-	-	-	-	-	-	-	-	+	+	+	+	-	-	+	-	-	+	+	-
n̩	+	-	-	-	-	-	-	-	-	-	+	-	+	+	+	-	-	-	-	-	+	+	-
tɸʰ	+	-	-	-	-	-	-	-	+	-	-	-	-	-	-	+	-	-	-	-	+	-	-
tʰ	+	-	-	-	-	-	-	-	+	-	-	-	-	-	+	+	-	-	-	-	+	-	-
dʰ	+	-	-	-	-	-	-	-	+	-	-	-	-	-	+	+	-	-	-	-	+	+	-
tsʰ	+	-	-	-	-	-	-	-	+	-	-	-	-	-	+	+	-	-	+	-	+	-	-
dzʰ	+	-	-	-	-	-	-	-	+	-	-	-	-	-	+	+	-	-	+	-	+	+	-
tʰ	+	-	-	-	-	-	-	-	+	-	-	-	+	-	+	+	-	-	-	-	+	-	-
nʰ, n̤	+	-	-	-	-	-	-	-	+	-	-	-	+	+	+	+	-	-	-	-	+	+	-
sʰ	+	-	-	-	-	-	-	-	+	-	-	+	-	-	+	+	-	-	+	-	+	-	-
z̤	+	-	-	-	-	-	-	-	+	-	-	+	-	-	+	+	-	-	+	-	+	+	-
r̥ʰ	+	-	-	-	-	-	-	-	+	-	-	+	-	+	+	+	-	-	-	-	+	-	-
rʰ	+	-	-	-	-	-	-	-	+	-	-	+	-	+	+	+	-	+	-	-	+	+	-
tː	+	-	-	-	-	-	-	+	-	-	-	-	-	-	+	-	-	-	-	-	+	-	-
nː	+	-	-	-	-	-	-	+	-	-	-	-	+	+	+	-	-	-	-	-	+	+	-
sː	+	-	-	-	-	-	-	+	-	-	-	+	-	-	+	-	-	-	+	-	+	-	-
rː, ɹː	+	-	-	-	-	-	-	+	-	-	-	+	-	+	+	-	-	+	-	-	+	+	-
t̪	+	-	-	-	-	-	+	-	-	-	-	-	-	-	+	-	-	-	-	-	+	-	-
d̪, d̻	+	-	-	-	-	-	+	-	-	-	-	-	-	-	+	-	-	-	-	-	+	+	-
t̪s̪, t̪s̪ʱ, t̻s̻	+	-	-	-	-	-	+	-	-	-	-	-	-	-	+	-	-	-	+	-	+	-	-
d̪z̪	+	-	-	-	-	-	+	-	-	-	-	-	-	-	+	-	-	-	+	-	+	+	-
ɗ̪	+	-	-	-	-	-	+	-	-	-	-	-	-	-	+	-	+	-	-	-	+	+	-
t̪	+	-	-	-	-	-	+	-	-	-	-	-	-	-	+	+	-	-	-	-	+	-	-
t̪’	+	-	-	-	-	-	+	-	-	-	-	-	-	-	+	+	+	-	-	-	+	-	-
t̪s̪’	+	-	-	-	-	-	+	-	-	-	-	-	-	-	+	+	+	-	+	-	+	-	-
ɾ̪	+	-	-	-	-	-	+	-	-	-	-	-	-	+	+	-	-	+	-	-	+	+	-
n̪t̪, t̪ⁿ	+	-	-	-	-	-	+	-	-	-	-	-	+	-	+	-	-	-	-	-	+	-	-
d̪	+	-	-	-	-	-	+	-	-	-	-	-	+	-	+	-	-	-	-	-	+	+	-
n̪t̪’	+	-	-	-	-	-	+	-	-	-	-	-	+	-	+	+	+	-	-	-	+	-	-
n̪, n̻	+	-	-	-	-	-	+	-	-	-	-	-	+	+	+	-	-	-	-	-	+	+	-
n̪’	+	-	-	-	-	-	+	-	-	-	-	-	+	+	+	+	+	-	-	-	+	+	-
s̪, s̻	+	-	-	-	-	-	+	-	-	-	-	+	-	-	+	-	-	-	+	-	+	-	-
z̪, z̻	+	-	-	-	-	-	+	-	-	-	-	+	-	-	+	-	-	-	+	-	+	+	-
s̪’	+	-	-	-	-	-	+	-	-	-	-	+	-	-	+	+	+	-	+	-	+	-	-
r̪	+	-	-	-	-	-	+	-	-	-	-	+	-	+	+	-	-	+	-	-	+	+	-
n̪z̪	+	-	-	-	-	-	+	-	-	-	-	+	+	-	+	-	-	-	+	-	+	+	-
t̪ʰ	+	-	-	-	-	-	+	-	+	-	-	-	-	-	+	+	-	-	-	-	+	-	-
d̪ʰ	+	-	-	-	-	-	+	-	+	-	-	-	-	-	+	+	-	-	-	-	+	+	-
t̪s̪ʰ	+	-	-	-	-	-	+	-	+	-	-	-	-	-	+	+	-	-	+	-	+	-	-
n̪ʰ	+	-	-	-	-	-	+	-	+	-	-	-	+	+	+	+	-	-	-	-	+	+	-
t̪ː	+	-	-	-	-	-	+	+	-	-	-	-	-	-	+	-	-	-	-	-	+	-	-
d̪ː	+	-	-	-	-	-	+	+	-	-	-	-	-	-	+	-	-	-	-	-	+	+	-
t̪s̪ː	+	-	-	-	-	-	+	+	-	-	-	-	-	-	+	-	-	-	+	-	+	-	-
n̪ː	+	-	-	-	-	-	+	+	-	-	-	-	+	+	+	-	-	-	-	-	+	+	-
s̪ː	+	-	-	-	-	-	+	+	-	-	-	+	-	-	+	-	-	-	+	-	+	-	-
z̪ː	+	-	-	-	-	-	+	+	-	-	-	+	-	-	+	-	-	-	+	-	+	+	-

	anterior	labial	back	high	ATR	lateral	distributed	LONG	spread	low	syllabic	continuant	nasal	sonorant	coronal	tense	constr	vocalic	strident	EXTRA	consonantal	voice	round
bɮ	+	-	-	-	-	+	-	-	-	-	-	-	-	-	-	-	-	-	-	-	+	+	-
tl, tɬ	+	-	-	-	-	+	-	-	-	-	-	-	-	-	+	-	-	-	-	-	+	-	-
dl	+	-	-	-	-	+	-	-	-	-	-	-	-	-	+	-	-	-	-	-	+	+	-
tl’, tɬ’	+	-	-	-	-	+	-	-	-	-	-	-	-	-	+	+	+	-	-	-	+	-	-
l̥	+	-	-	-	-	+	-	-	-	-	-	-	-	+	+	-	-	-	-	-	+	-	-
l̀, l̟, ɺ̀, ɾˡ	+	-	-	-	-	+	-	-	-	-	-	-	-	+	+	-	-	+	-	-	+	+	-
l, ɺ̤, ʔl	+	-	-	-	-	+	-	-	-	-	-	-	-	+	+	+	-	+	-	-	+	+	-
l’	+	-	-	-	-	+	-	-	-	-	-	-	-	+	+	+	+	+	-	-	+	+	-
tɬ	+	-	-	-	-	+	-	-	-	-	-	-	+	-	+	-	-	-	-	-	+	-	-
dl, dɮ	+	-	-	-	-	+	-	-	-	-	-	-	+	-	+	-	-	-	-	-	+	+	-
dl’	+	-	-	-	-	+	-	-	-	-	-	-	+	-	+	+	+	-	-	-	+	+	-
mnl	+	-	-	-	-	+	-	-	-	-	-	-	+	+	-	-	-	-	-	-	+	+	-
l̃	+	-	-	-	-	+	-	-	-	-	-	-	+	+	+	-	-	+	-	-	+	+	-
ɬ	+	-	-	-	-	+	-	-	-	-	-	+	-	-	+	-	-	-	-	-	+	-	-
ɮ	+	-	-	-	-	+	-	-	-	-	-	+	-	-	+	-	-	-	-	-	+	+	-
ɬ̀	+	-	-	-	-	+	-	-	-	-	-	+	-	-	+	+	-	-	-	-	+	-	-
ɬ’	+	-	-	-	-	+	-	-	-	-	-	+	-	-	+	+	+	-	-	-	+	-	-
ɬ, ᵐɬ	+	-	-	-	-	+	-	-	-	-	-	+	+	-	+	-	-	-	-	-	+	-	-
ɮ	+	-	-	-	-	+	-	-	-	-	-	+	+	-	+	-	-	-	-	-	+	+	-
l̩	+	-	-	-	-	+	-	-	-	-	+	-	-	+	+	-	-	+	-	-	+	+	-
tlʰ, tɬʰ	+	-	-	-	-	+	-	-	+	-	-	-	-	-	+	+	-	-	-	-	+	-	-
l̥ʰ	+	-	-	-	-	+	-	-	+	-	-	-	-	+	+	+	-	-	-	-	+	-	-
l̀ʰ, l̤	+	-	-	-	-	+	-	-	+	-	-	-	-	+	+	+	-	+	-	-	+	+	-
lː	+	-	-	-	-	+	-	+	-	-	-	-	-	+	+	-	-	+	-	-	+	+	-
l̪θ	+	-	-	-	-	+	+	-	-	-	-	-	-	-	+	-	-	-	-	-	+	-	-
l̪	+	-	-	-	-	+	+	-	-	-	-	-	-	+	+	-	-	+	-	-	+	+	-
l̪’	+	-	-	-	-	+	+	-	-	-	-	-	-	+	+	+	+	+	-	-	+	+	-
l̪ʰ	+	-	-	-	-	+	+	-	+	-	-	-	-	+	+	+	-	+	-	-	+	+	-
l̪ː	+	-	-	-	-	+	+	+	-	-	-	-	-	+	+	-	-	+	-	-	+	+	-
tʲ	+	-	-	+	-	-	-	-	-	-	-	-	-	-	+	-	-	-	-	-	+	-	-
dʲ	+	-	-	+	-	-	-	-	-	-	-	-	-	-	+	-	-	-	-	-	+	+	-
tsʲ	+	-	-	+	-	-	-	-	-	-	-	-	-	-	+	-	-	-	+	-	+	-	-
dzʲ	+	-	-	+	-	-	-	-	-	-	-	-	-	-	+	-	-	-	+	-	+	+	-
tʲ’	+	-	-	+	-	-	-	-	-	-	-	-	-	-	+	+	+	-	-	-	+	-	-
ɾʲ	+	-	-	+	-	-	-	-	-	-	-	-	-	+	+	-	-	+	-	-	+	+	-
dʲ	+	-	-	+	-	-	-	-	-	-	-	-	+	-	+	-	-	-	-	-	+	+	-
nʲ, n̟ʲ	+	-	-	+	-	-	-	-	-	-	-	-	+	+	+	-	-	-	-	-	+	+	-
sʲ	+	-	-	+	-	-	-	-	-	-	-	+	-	-	+	-	-	-	+	-	+	-	-
zʲ	+	-	-	+	-	-	-	-	-	-	-	+	-	-	+	-	-	-	+	-	+	+	-
rʲ, r̟ʲ	+	-	-	+	-	-	-	-	-	-	-	+	-	+	+	-	-	+	-	-	+	+	-
tʲʰ	+	-	-	+	-	-	-	-	+	-	-	-	-	-	+	+	-	-	-	-	+	-	-
tsjʰ	+	-	-	+	-	-	-	-	+	-	-	-	-	-	+	+	-	-	+	-	+	-	-
t̪ʲ	+	-	-	+	-	-	+	-	-	-	-	-	-	-	+	-	-	-	-	-	+	-	-
d̪ʲ	+	-	-	+	-	-	+	-	-	-	-	-	-	-	+	-	-	-	-	-	+	+	-
t̪s̪ʲ	+	-	-	+	-	-	+	-	-	-	-	-	-	-	+	-	-	-	+	-	+	-	-
d̪z̪ʲ	+	-	-	+	-	-	+	-	-	-	-	-	-	-	+	-	-	-	+	-	+	+	-
n̪ʲ	+	-	-	+	-	-	+	-	-	-	-	-	+	+	+	-	-	-	-	-	+	+	-
s̪ʲ	+	-	-	+	-	-	+	-	-	-	-	+	-	-	+	-	-	-	+	-	+	-	-
z̪ʲ	+	-	-	+	-	-	+	-	-	-	-	+	-	-	+	-	-	-	+	-	+	+	-
r̪ʲ	+	-	-	+	-	-	+	-	-	-	-	+	-	+	+	-	-	+	-	-	+	+	-
l̀ʲ, l̟ʲ	+	-	-	+	-	+	-	-	-	-	-	-	-	+	+	-	-	+	-	-	+	+	-
l̪ʲ	+	-	-	+	-	+	+	-	-	-	-	-	-	+	+	-	-	+	-	-	+	+	-
tˤ	+	-	+	-	-	-	-	-	-	+	-	-	-	-	+	-	-	-	-	-	+	-	-
dˤ	+	-	+	-	-	-	-	-	-	+	-	-	-	-	+	-	-	-	-	-	+	+	-
ɾˤ	+	-	+	-	-	-	-	-	-	+	-	-	-	+	+	-	-	+	-	-	+	+	-
nˤ	+	-	+	-	-	-	-	-	-	+	-	-	+	+	+	-	-	-	-	-	+	+	-

	anterior	labial	back	high	ATR	lateral	distributed	LONG	spread	low	syllabic	continuant	nasal	sonorant	coronal	tense	constr	vocalic	strident	EXTRA	consonantal	voice	round
ðˤ	+	-	+	-	-	-	-	-	-	+	-	+	-	-	+	-	-	-	-	-	+	+	-
sˤ	+	-	+	-	-	-	-	-	-	+	-	+	-	-	+	-	-	-	+	-	+	-	-
zˤ	+	-	+	-	-	-	-	-	-	+	-	+	-	-	+	-	-	-	+	-	+	+	-
rˤ	+	-	+	-	-	-	-	-	-	+	-	+	-	+	+	-	-	+	-	-	+	+	-
nˤː	+	-	+	-	-	-	-	+	-	+	-	-	+	+	+	-	-	-	-	-	+	+	-
rˤː	+	-	+	-	-	-	-	+	-	+	-	+	-	+	+	-	-	+	-	-	+	+	-
t̪ˤ	+	-	+	-	-	-	+	-	-	+	-	-	-	-	+	-	-	-	-	-	+	-	-
d̪ˤ	+	-	+	-	-	-	+	-	-	+	-	-	-	-	+	-	-	-	-	-	+	+	-
ɾ̪ˤ	+	-	+	-	-	-	+	-	-	+	-	-	-	+	+	-	-	+	-	-	+	+	-
s̪ˤ, ʂˤ	+	-	+	-	-	-	+	-	-	+	-	+	-	-	+	-	-	-	+	-	+	-	-
z̪ˤ	+	-	+	-	-	-	+	-	-	+	-	+	-	-	+	-	-	-	+	-	+	+	-
t̪ˤː	+	-	+	-	-	-	+	+	-	+	-	-	-	-	+	-	-	-	-	-	+	-	-
d̪ˤː	+	-	+	-	-	-	+	+	-	+	-	-	-	-	+	-	-	-	-	-	+	+	-
s̪ˤː	+	-	+	-	-	-	+	+	-	+	-	+	-	-	+	-	-	-	+	-	+	-	-
z̪ˤː	+	-	+	-	-	-	+	+	-	+	-	+	-	-	+	-	-	-	+	-	+	+	-
l̪ˤ	+	-	+	-	-	+	+	-	-	+	-	-	-	+	+	-	-	+	-	-	+	+	-
l̪ˤː	+	-	+	-	-	+	+	+	-	+	-	-	-	+	+	-	-	+	-	-	+	+	-
tˠ, !, !d, !q, !r, !s, !t, !x, !z, !ʃ, !ʒ	+	-	+	+	-	-	-	-	-	-	-	-	-	-	+	-	-	-	-	-	+	-	-
dˠ, g!, g!kx, g!x	+	-	+	+	-	-	-	-	-	-	-	-	-	-	+	-	-	-	-	-	+	+	-
!kxʔ, !qʔ, !ʔ	+	-	+	+	-	-	-	-	-	-	-	-	-	-	+	+	+	-	-	-	+	-	-
m!G, nk!, n!, !n, !n̥	+	-	+	+	-	-	-	-	-	-	-	-	+	-	+	-	-	-	-	-	+	-	-
ng!	+	-	+	+	-	-	-	-	-	-	-	-	+	-	+	-	-	-	-	-	+	+	-
ʔ!n, ʔΘn	+	-	+	+	-	-	-	-	-	-	-	-	+	-	+	+	+	-	-	-	+	-	-
ðˠ	+	-	+	+	-	-	-	-	-	-	-	+	-	-	+	-	-	-	-	-	+	+	-
sˠ	+	-	+	+	-	-	-	-	-	-	-	+	-	-	+	-	-	-	+	-	+	-	-
!qʰ	+	-	+	+	-	-	-	-	+	-	-	-	-	-	+	-	-	-	-	-	+	-	-
!ʰ	+	-	+	+	-	-	-	-	+	-	-	-	-	-	+	+	-	-	-	-	+	-	-
g!ʰ	+	-	+	+	-	-	-	-	+	-	-	-	-	-	+	+	-	-	-	-	+	+	-
nkǀ, ǀ, ǀq, ǀx	+	-	+	+	-	-	+	-	-	-	-	-	-	-	+	-	-	-	-	-	+	-	-
gǀ, gǀkx, gǀx	+	-	+	+	-	-	+	-	-	-	-	-	-	-	+	-	-	-	-	-	+	+	-
ǀkxʔ, ǀqʔ, ǀʔ	+	-	+	+	-	-	+	-	-	-	-	-	-	-	+	+	+	-	-	-	+	-	-
mǀG, nǀ, ǀ, ǀn, ǀn̥	+	-	+	+	-	-	+	-	-	-	-	-	+	-	+	-	-	-	-	-	+	-	-
ngǀ	+	-	+	+	-	-	+	-	-	-	-	-	+	-	+	-	-	-	-	-	+	+	-
ʔǀn	+	-	+	+	-	-	+	-	-	-	-	-	+	-	+	+	+	-	-	-	+	-	-
ǀqʰ	+	-	+	+	-	-	+	-	+	-	-	-	-	-	+	-	-	-	-	-	+	-	-
ǀʰ	+	-	+	+	-	-	+	-	+	-	-	-	-	-	+	+	-	-	-	-	+	-	-
gǀʰ	+	-	+	+	-	-	+	-	+	-	-	-	-	-	+	+	-	-	-	-	+	+	-
ǁ, ǁq, ǁx	+	-	+	+	-	+	-	-	-	-	-	-	-	-	+	-	-	-	-	-	+	-	-
l!, gǁ, gǁkx, gǁx	+	-	+	+	-	+	-	-	-	-	-	-	-	-	+	-	-	-	-	-	+	+	-
ǁkxʔ, ǁqʔ, ǁʔ	+	-	+	+	-	+	-	-	-	-	-	-	-	-	+	+	+	-	-	-	+	-	-
lˠ	+	-	+	+	-	+	-	-	-	-	-	-	-	+	+	-	-	+	-	-	+	+	-
nǁ	+	-	+	+	-	+	-	-	-	-	-	-	+	-	+	-	-	-	-	-	+	-	-
mǁG, ngǁ, ǁn, ǁn̥	+	-	+	+	-	+	-	-	-	-	-	-	+	-	+	-	-	-	-	-	+	+	-
ʔǁn	+	-	+	+	-	+	-	-	-	-	-	-	+	-	+	+	+	-	-	-	+	-	-

	anterior	labial	back	high	ATR	lateral	distributed	LONG	spread	low	syllabic	continuant	nasal	sonorant	coronal	tense	constr	vocalic	strident	EXTRA	consonantal	voice	round
ǁqʰ	+	-	+	+	-	+	-	-	+	-	-	-	-	-	+	-	-	-	-	-	+	-	-
ǁʰ	+	-	+	+	-	+	-	-	+	-	-	-	-	-	+	+	-	-	-	-	+	-	-
gǁʰ	+	-	+	+	-	+	-	-	+	-	-	-	-	-	+	+	-	-	-	-	+	+	-
s͡kʷ	+	+	-	-	-	-	-	-	-	-	-	-	-	-	-	-	-	-	-	-	+	-	+
pf, pv	+	+	-	-	-	-	-	-	-	-	-	-	-	-	-	-	-	-	+	-	+	-	-
ts͡kʷ	+	+	-	-	-	-	-	-	-	-	-	-	-	-	-	-	-	-	+	-	+	-	+
bv	+	+	-	-	-	-	-	-	-	-	-	-	-	-	-	-	-	-	+	-	+	+	-
tʷ	+	+	-	-	-	-	-	-	-	-	-	-	-	-	+	-	-	-	-	-	+	-	+
dʷ	+	+	-	-	-	-	-	-	-	-	-	-	-	-	+	-	-	-	-	-	+	+	+
tsʷ	+	+	-	-	-	-	-	-	-	-	-	-	-	-	+	-	-	-	+	-	+	-	+
dzʷ	+	+	-	-	-	-	-	-	-	-	-	-	-	-	+	-	-	-	+	-	+	+	+
ɗʷ	+	+	-	-	-	-	-	-	-	-	-	-	-	-	+	-	+	-	-	-	+	+	+
t'ʷ	+	+	-	-	-	-	-	-	-	-	-	-	-	-	+	+	+	-	-	-	+	-	+
ts'ʷ	+	+	-	-	-	-	-	-	-	-	-	-	-	-	+	+	+	-	+	-	+	-	+
vɾ	+	+	-	-	-	-	-	-	-	-	-	-	-	+	-	-	-	+	-	-	+	+	-
f, v̥, ɱ̊f	+	+	-	-	-	-	-	-	-	-	-	+	-	-	-	-	-	-	+	-	+	-	-
fʷ	+	+	-	-	-	-	-	-	-	-	-	+	-	-	-	-	-	-	+	-	+	-	+
v	+	+	-	-	-	-	-	-	-	-	-	+	-	-	-	-	-	-	+	-	+	+	-
f	+	+	-	-	-	-	-	-	-	-	-	+	-	-	-	+	-	-	+	-	+	-	-
v	+	+	-	-	-	-	-	-	-	-	-	+	-	-	-	+	-	-	+	-	+	+	-
f'	+	+	-	-	-	-	-	-	-	-	-	+	-	-	-	+	+	-	+	-	+	-	-
sʷ	+	+	-	-	-	-	-	-	-	-	-	+	-	-	+	-	-	-	+	-	+	-	+
zʷ	+	+	-	-	-	-	-	-	-	-	-	+	-	-	+	-	-	-	+	-	+	+	+
ʃ'ʷ	+	+	-	-	-	-	-	-	-	-	-	+	-	-	+	+	+	-	+	-	+	-	+
ʋ*, ʋ	+	+	-	-	-	-	-	-	-	-	-	+	-	+	-	-	-	-	-	-	-	+	-
v̌	+	+	-	-	-	-	-	-	-	-	-	+	-	+	-	-	-	+	-	-	+	+	-
ʋ, ʋʰ	+	+	-	-	-	-	-	-	-	-	-	+	-	+	-	+	-	-	-	-	-	+	-
rʷ	+	+	-	-	-	-	-	-	-	-	-	+	-	+	+	-	-	+	-	-	+	+	+
ᵐf	+	+	-	-	-	-	-	-	-	-	-	+	+	-	-	-	-	-	+	-	+	-	-
nzʷ	+	+	-	-	-	-	-	-	-	-	-	+	+	-	+	-	-	-	+	-	+	+	+
ʋ̃	+	+	-	-	-	-	-	-	-	-	-	+	+	+	-	-	-	-	-	-	-	+	-
pfʰ	+	+	-	-	-	-	-	-	+	-	-	-	-	-	-	+	-	-	+	-	+	-	-
tʰʷ	+	+	-	-	-	-	-	-	+	-	-	-	-	-	+	+	-	-	-	-	+	-	+
tswʰ, tsʰʷ	+	+	-	-	-	-	-	-	+	-	-	-	-	-	+	+	-	-	+	-	+	-	+
vʰ, v̤	+	+	-	-	-	-	-	-	+	-	-	+	-	-	-	+	-	-	+	-	+	+	-
fː	+	+	-	-	-	-	-	+	-	-	-	+	-	-	-	-	-	-	+	-	+	-	-
b̥, p*, p, pɸ, pʱ	+	+	-	-	-	-	+	-	-	-	-	-	-	-	-	-	-	-	-	-	+	-	-
pʷ	+	+	-	-	-	-	+	-	-	-	-	-	-	-	-	-	-	-	-	-	+	-	+
b*, b, b̂	+	+	-	-	-	-	+	-	-	-	-	-	-	-	-	-	-	-	-	-	+	+	-
bʷ	+	+	-	-	-	-	+	-	-	-	-	-	-	-	-	-	-	-	-	-	+	+	+
ɓ̥	+	+	-	-	-	-	+	-	-	-	-	-	-	-	-	-	+	-	-	-	+	-	-
ɓ̊	+	+	-	-	-	-	+	-	-	-	-	-	-	-	-	-	+	-	-	-	+	+	-
ɓʷ	+	+	-	-	-	-	+	-	-	-	-	-	-	-	-	-	+	-	-	-	+	+	+
p, p̀	+	+	-	-	-	-	+	-	-	-	-	-	-	-	-	+	-	-	-	-	+	-	-
b, b̏	+	+	-	-	-	-	+	-	-	-	-	-	-	-	-	+	-	-	-	-	+	+	-
p', ʔp	+	+	-	-	-	-	+	-	-	-	-	-	-	-	-	+	+	-	-	-	+	-	-
pʷ'	+	+	-	-	-	-	+	-	-	-	-	-	-	-	-	+	+	-	-	-	+	-	+
b', ɓ, ʔb	+	+	-	-	-	-	+	-	-	-	-	-	-	-	-	+	+	-	-	-	+	+	-
t̪ʷ	+	+	-	-	-	-	+	-	-	-	-	-	-	-	+	-	-	-	-	-	+	-	+
d̪z̪ʷ	+	+	-	-	-	-	+	-	-	-	-	-	-	-	+	-	-	-	+	-	+	+	+
mb̥, pᵐ, ᵐp	+	+	-	-	-	-	+	-	-	-	-	-	+	-	-	-	-	-	-	-	+	-	-
ᵐb	+	+	-	-	-	-	+	-	-	-	-	-	+	-	-	-	-	-	-	-	+	+	-
ᵐbʷ	+	+	-	-	-	-	+	-	-	-	-	-	+	-	-	-	-	-	-	-	+	+	+
ᵑb	+	+	-	-	-	-	+	-	-	-	-	-	+	-	-	+	-	-	-	-	+	+	-

	anterior	labial	back	high	ATR	lateral	distributed	LONG	spread	low	syllabic	continuant	nasal	sonorant	coronal	tense	constr	vocalic	strident	EXTRA	consonantal	voice	round
ᵐp’	+	+	-	-	-	-	+	-	-	-	-	-	+	-	-	+	+	-	-	-	+	-	-
m̥	+	+	-	-	-	-	+	-	-	-	-	-	+	+	-	-	-	-	-	-	+	-	-
m, m̂	+	+	-	-	-	-	+	-	-	-	-	-	+	+	-	-	-	-	-	-	+	+	-
mʷ	+	+	-	-	-	-	+	-	-	-	-	-	+	+	-	-	-	-	-	-	+	+	+
m̏	+	+	-	-	-	-	+	-	-	-	-	-	+	+	-	+	-	-	-	-	+	+	-
m’, ʔm	+	+	-	-	-	-	+	-	-	-	-	-	+	+	-	+	+	-	-	-	+	+	-
ɸ	+	+	-	-	-	-	+	-	-	-	-	+	-	-	-	-	-	-	-	-	+	-	-
β	+	+	-	-	-	-	+	-	-	-	-	+	-	-	-	-	-	-	-	-	+	+	-
mβ, βm, ᵐv	+	+	-	-	-	-	+	-	-	-	-	+	+	-	-	-	-	-	-	-	+	+	-
m̩	+	+	-	-	-	-	+	-	-	-	+	-	+	+	-	-	-	-	-	-	+	+	-
pʰ	+	+	-	-	-	-	+	-	+	-	-	-	-	-	-	+	-	-	-	-	+	-	-
pʰʷ	+	+	-	-	-	-	+	-	+	-	-	-	-	-	-	+	-	-	-	-	+	-	+
bʰ	+	+	-	-	-	-	+	-	+	-	-	-	-	-	-	+	-	-	-	-	+	+	-
t̪s̪ʰʷ	+	+	-	-	-	-	+	-	+	-	-	-	-	-	+	+	-	-	+	-	+	-	+
ᵐpʰ	+	+	-	-	-	-	+	-	+	-	-	-	+	-	-	+	-	-	-	-	+	-	-
m̥ʰ	+	+	-	-	-	-	+	-	+	-	-	-	+	+	-	+	-	-	-	-	+	-	-
mʰ, m̤	+	+	-	-	-	-	+	-	+	-	-	-	+	+	-	+	-	-	-	-	+	+	-
pː	+	+	-	-	-	-	+	+	-	-	-	-	-	-	-	-	-	-	-	-	+	-	-
bː	+	+	-	-	-	-	+	+	-	-	-	-	-	-	-	-	-	-	-	-	+	+	-
mː	+	+	-	-	-	-	+	+	-	-	-	-	+	+	-	-	-	-	-	-	+	+	-
lʷ	+	+	-	-	-	+	-	-	-	-	-	-	-	+	+	-	-	+	-	-	+	+	+
fˡ	+	+	-	-	-	+	-	-	-	-	-	+	-	-	-	-	-	-	+	-	+	-	-
vˡ	+	+	-	-	-	+	-	-	-	-	-	+	-	-	-	-	-	-	+	-	+	+	-
ɬʷ	+	+	-	-	-	+	-	-	-	-	-	+	-	-	+	-	-	-	-	-	+	-	+
pˡ	+	+	-	-	-	+	+	-	-	-	-	-	-	-	-	-	-	-	-	-	+	-	-
bˡ	+	+	-	-	-	+	+	-	-	-	-	-	-	-	-	-	-	-	-	-	+	+	-
mˡ	+	+	-	-	-	+	+	-	-	-	-	-	+	+	-	-	-	-	-	-	+	+	-
fʲ	+	+	-	+	-	-	-	-	-	-	-	+	-	-	-	-	-	-	+	-	+	-	-
vʲ	+	+	-	+	-	-	-	-	-	-	-	+	-	-	-	-	-	-	+	-	+	+	-
sɥ	+	+	-	+	-	-	-	-	-	-	-	+	-	-	+	-	-	-	+	-	+	-	+
ʋʲ	+	+	-	+	-	-	-	-	-	-	-	+	-	+	-	-	-	-	-	-	-	+	-
ṽʲ	+	+	-	+	-	-	-	-	-	-	-	+	+	-	-	-	-	-	+	-	+	+	-
pʲ	+	+	-	+	-	-	+	-	-	-	-	-	-	-	-	-	-	-	-	-	+	-	-
bʲ	+	+	-	+	-	-	+	-	-	-	-	-	-	-	-	-	-	-	-	-	+	+	-
ᵐbʲ	+	+	-	+	-	-	+	-	-	-	-	-	+	-	-	-	-	-	-	-	+	+	-
mʲ	+	+	-	+	-	-	+	-	-	-	-	-	+	+	-	-	-	-	-	-	+	+	-
pʲʰ	+	+	-	+	-	-	+	-	+	-	-	-	-	-	-	+	-	-	-	-	+	-	-
lɥ	+	+	-	+	-	+	-	-	-	-	-	-	-	+	+	-	-	+	-	-	+	+	+
o̥	+	+	+	-	-	-	-	-	-	-	-	+	-	+	-	-	-	-	-	-	-	+	-
f̂ˤ	+	+	+	-	-	-	-	-	-	+	-	+	-	-	-	-	-	-	+	-	+	-	-
vˤ	+	+	+	-	-	-	-	-	-	+	-	+	-	-	-	-	-	-	+	-	+	+	-
pˤ	+	+	+	-	-	-	+	-	-	+	-	-	-	-	-	-	-	-	-	-	+	-	-
bˤ	+	+	+	-	-	-	+	-	-	+	-	-	-	-	-	-	-	-	-	-	+	+	-
p’ˤ	+	+	+	-	-	-	+	-	-	+	-	-	-	-	-	+	+	-	-	-	+	-	-
mˤ	+	+	+	-	-	-	+	-	-	+	-	-	+	+	-	-	-	-	-	-	+	+	-
k͡p̂, k͡p, k͡pʲ, g͡p	+	+	+	+	-	-	-	-	-	-	-	-	-	-	-	-	-	-	-	-	+	-	-
k͡pʷ	+	+	+	+	-	-	-	-	-	-	-	-	-	-	-	-	-	-	-	-	+	-	+
ĝ͡b, g͡b, g͡bʲ	+	+	+	+	-	-	-	-	-	-	-	-	-	-	-	-	-	-	-	-	+	+	-
g͡bʷ	+	+	+	+	-	-	-	-	-	-	-	-	-	-	-	-	-	-	-	-	+	+	+
g͡ɓ	+	+	+	+	-	-	-	-	-	-	-	-	-	-	-	-	+	-	-	-	+	+	-
k͡p	+	+	+	+	-	-	-	-	-	-	-	-	-	-	-	+	-	-	-	-	+	-	-
g͡b	+	+	+	+	-	-	-	-	-	-	-	-	-	-	-	+	-	-	-	-	+	+	-

	anterior	labial	back	high	ATR	lateral	distributed	LONG	spread	low	syllabic	continuant	nasal	sonorant	coronal	tense	constr	vocalic	strident	EXTRA	consonantal	voice	round
g͡ɓ	+	+	+	+	-	-	-	-	-	-	-	-	-	-	-	+	+	-	-	-	+	+	-
ᵐk͡p	+	+	+	+	-	-	-	-	-	-	-	-	+	-	-	-	-	-	-	-	+	-	-
ᵐg͡b, ᵑg͡b, ᵑᵐg͡b	+	+	+	+	-	-	-	-	-	-	-	-	+	-	-	-	-	-	-	-	+	+	-
ŋ͡m, ŋ͡mʲ	+	+	+	+	-	-	-	-	-	-	-	-	+	+	-	-	-	-	-	-	+	+	-
w*, w, wʱ, wʲ	+	+	+	+	-	-	-	-	-	-	-	+	-	+	-	-	-	-	-	-	-	+	-
w	+	+	+	+	-	-	-	-	-	-	-	+	-	+	-	+	-	-	-	-	-	+	-
wʰ	+	+	+	+	-	-	-	-	-	-	-	+	-	+	-	+	-	-	-	-	-	+	+
ʔw	+	+	+	+	-	-	-	-	-	-	-	+	-	+	-	+	+	-	-	-	-	+	-
w’	+	+	+	+	-	-	-	-	-	-	-	+	-	+	-	+	+	-	-	-	-	+	+
w̃	+	+	+	+	-	-	-	-	-	-	-	+	+	+	-	-	-	-	-	-	-	+	-
wˤ	+	+	+	+	-	-	-	-	-	+	-	+	-	+	-	-	-	-	-	-	-	+	-
p͡kʰ	+	+	+	+	-	-	-	-	+	-	-	-	-	-	-	+	-	-	-	-	+	-	-
ʍ	+	+	+	+	-	-	-	-	+	-	-	+	-	+	-	-	-	-	-	-	-	-	-
ʍ	+	+	+	+	-	-	-	-	+	-	-	+	-	+	-	+	-	-	-	-	-	-	-
wː	+	+	+	+	-	-	-	+	-	-	-	+	-	+	-	-	-	-	-	-	-	+	-
pˠ, ʘ, ʘq, ʘx	+	+	+	+	-	-	+	-	-	-	-	-	-	-	-	-	-	-	-	-	+	-	-
gʘ, gʘkx, gʘx	+	+	+	+	-	-	+	-	-	-	-	-	-	-	-	-	-	-	-	-	+	+	-
ɓ̥ˠ	+	+	+	+	-	-	+	-	-	-	-	-	-	-	-	-	+	-	-	-	+	-	-
ʘ̊kxʔ, ʘqʔ, ʘʔ	+	+	+	+	-	-	+	-	-	-	-	-	-	-	-	+	+	-	-	-	+	-	-
mʘG, ʘn, ʘn̥	+	+	+	+	-	-	+	-	-	-	-	-	+	-	-	-	-	-	-	-	+	-	-
mˠ	+	+	+	+	-	-	+	-	-	-	-	-	+	+	-	-	-	-	-	-	+	+	-
ʘqʰ	+	+	+	+	-	-	+	-	+	-	-	-	-	-	-	-	-	-	-	-	+	-	-
ʘʰ	+	+	+	+	-	-	+	-	+	-	-	-	-	-	-	+	-	-	-	-	+	-	-
gʘʰ	+	+	+	+	-	-	+	-	+	-	-	-	-	-	-	+	-	-	-	-	+	+	-
lˠʷ	+	+	+	+	-	+	-	-	-	-	-	-	-	+	+	-	-	+	-	-	+	+	+
